# Elevated plasma and CSF neurofilament light chain concentrations are stabilized in response to mutant huntingtin lowering in the brains of Huntington’s disease mice

**DOI:** 10.1186/s40035-024-00443-8

**Published:** 2024-10-08

**Authors:** Nicholas S. Caron, Lauren M. Byrne, Fanny L. Lemarié, Jeffrey N. Bone, Amirah E.-E. Aly, Seunghyun Ko, Christine Anderson, Lorenzo L. Casal, Austin M. Hill, David J. Hawellek, Peter McColgan, Edward J. Wild, Blair R. Leavitt, Michael R. Hayden

**Affiliations:** 1 Centre for Molecular Medicine and Therapeutics, Vancouver, BC V5Z 4H4 Canada; 2 BC Children’s Hospital Research Institute, Vancouver, BC V5Z 4H4 Canada; 3 Department of Medical Genetics, University of British Columbia, Vancouver, BC V6T 1Z3 Canada; 4 UCL Huntington’s Disease Centre, University College London Queen Square Institute of Neurology, London, WC1N 3BG UK; 5 Department of Statistics, University of British Columbia, Vancouver, BC V6T 1Z2 Canada; 6 Roche Pharma Research and Early Development, Roche Innovation Center Basel, F. Hoffmann-La Roche Ltd., Grenzacherstrasse 124, 4070 Basel, Switzerland; 7 Roche Products Ltd., Welwyn Garden City, AL7 1TW United Kingdom

**Keywords:** Neurofilament light chain, Response biomarker, Huntington's disease, Huntingtin lowering, Antisense oligonucleotide, Cerebrospinal fluid, Plasma, Biofluids

## Abstract

**Background:**

Therapeutic approaches aimed at lowering toxic mutant huntingtin (mHTT) levels in the brain can reverse disease phenotypes in animal models of Huntington's disease (HD) and are currently being evaluated in clinical trials. Sensitive and dynamic response biomarkers are needed to assess the efficacy of such candidate therapies. Neurofilament light chain (NfL) is a biomarker of neurodegeneration that increases in cerebrospinal fluid (CSF) and blood with progression of HD. However, it remains unknown whether NfL in biofluids could serve as a response biomarker for assessing the efficacy of disease-modifying therapies for HD.

**Methods:**

Longitudinal plasma and cross-sectional CSF samples were collected from the YAC128 transgenic mouse model of HD and wild-type (WT) littermate control mice throughout the natural history of disease. Additionally, biofluids were collected from YAC128 mice following intracerebroventricular administration of an antisense oligonucleotide (ASO) targeting the mutant *HTT* transgene (HTT ASO), at ages both before and after the onset of disease phenotypes. NfL concentrations in plasma and CSF were quantified using ultrasensitive single-molecule array technology.

**Results:**

Plasma and CSF NfL concentrations were significantly elevated in YAC128 compared to WT littermate control mice from 9 months of age. Treatment of YAC128 mice with either 15 or 50 µg HTT ASO resulted in a dose-dependent, allele-selective reduction of mHTT throughout the brain at a 3-month interval, which was sustained with high-dose HTT ASO treatment for up to 6 months. Lowering of brain mHTT prior to the onset of regional brain atrophy and HD-like motor deficits in this model had minimal effect on plasma NfL at either dose, but led to a dose-dependent reduction of CSF NfL. In contrast, initiating mHTT lowering in the brain after the onset of neuropathological and behavioural phenotypes in YAC128 mice resulted in a dose-dependent stabilization of NfL increases in both plasma and CSF.

**Conclusions:**

Our data provide evidence that the response of NfL in biofluids is influenced by the magnitude of mHTT lowering in the brain and the timing of intervention, suggesting that NfL may serve as a promising exploratory response biomarker for HD.

**Supplementary Information:**

The online version contains supplementary material available at 10.1186/s40035-024-00443-8.

## Background

Huntington’s disease (HD) is a fatal neurodegenerative disorder with no effective disease-modifying treatment. HD is caused by a CAG repeat expansion mutation in the *HTT* gene that codes for an abnormal polyglutamine tract in the huntingtin (HTT) protein [1]. Polyglutamine-expanded mutant HTT (mHTT) disrupts multiple cellular processes and pathways (reviewed in [2]), leading to progressive degeneration of selected neuronal populations in the basal ganglia and cerebral cortex [3–11].

Targeting a proximal cause of HD pathogenesis by lowering levels of mHTT in the central nervous system (CNS) has emerged as a promising therapeutic approach for HD. Preclinical studies in HD rodent models have demonstrated that pharmacological mHTT lowering in the CNS can prevent HD-like behavioural deficits and progressive neurological phenotypes, including regional forebrain atrophy and neurodegeneration [12–14]. These findings have accelerated clinical development of multiple HTT-lowering modalities, and have led to the therapeutic trials evaluating antisense oligonucleotides (ASOs) [15–20], RNA interference-based approaches [21, 22] and small molecules [23, 24] for HD. However, there remains a need to identify sensitive and objective biomarkers that change in a predictable manner in response to intervention to provide evidence of therapeutic efficacy.

Fluid biomarkers (or wet biomarkers) offer the potential to non-invasively and objectively monitor pathophysiological alterations in the CNS associated with HD. Sensitive and dynamic molecular biomarkers that reflect altered neuronal health, neurodegeneration or other underlying pathogenic processes could be used to detect early pathological changes in premanifest disease. They may also be useful to complement existing clinical measures and imaging biomarkers for monitoring disease progression and assessing therapeutic efficacy of candidate therapies in clinical trials for HD.

Multiple protein biomarkers have been identified in CSF and/or blood that are altered in HD mutation carriers and correlate with clinical measures of disease, including mHTT [25–28], neurofilament light chain (NfL) [27–33], proenkephalin [32, 34], prodynorphin [32, 35], microtubule-associated protein tau [36–38], and YKL-40 (chitinase-3-like protein 1) [39, 40]. Moreover, we recently defined specific combinations of CSF biomarkers that could be used to reliably stratify individuals based on HD severity [32].

Neurofilaments are a family of neuron-specific intermediate filaments that are key structural components of the axonal cytoskeleton. Upon axonal damage or neurodegeneration, NfL is released from neurons into the extracellular space where it is cleared into the CSF, and subsequently to the blood [41]. Elevated concentrations of NfL in biofluids have been reported in several neurological disorders, including HD [27–33, 42], spinocerebellar ataxias [43–45], spinal muscular atrophy (SMA) type 1 [46], amyotrophic lateral sclerosis (ALS) [47, 48], Parkinson’s disease [49, 50], and Alzheimer’s disease [51, 52]. Increased biofluid NfL concentrations have also been reported in multiple sclerosis (MS) [53–58], traumatic brain injury [59–61] and individuals with HIV infection [62].

In HD, concentrations of NfL in CSF and blood increase with disease severity and are associated with clinical measures of cognitive and motor function as well as imaging measures of regional brain atrophy [27, 29]. Moreover, NfL levels in biofluids are a strong prognostic biomarker of disease onset, clinical progression and brain atrophy in people with HD [28, 29, 31]. Notably, elevated CSF and plasma NfL are one of the earliest measurable pathological alterations in HD [27], with significant increases reported in premanifest HD mutation carriers approximately 24 years before predicted disease onset [42]. These findings suggest that increased NfL levels in blood and CSF reflect early perturbations to neuronal health in the brain that may precede neurodegeneration and subsequent cell loss. Consistent with data from HD mutation carriers, elevated NfL concentrations in biofluids have been reported in both transgenic (eg. YAC128) and knock-in (eg. zQ175) mouse models of HD [63–65].

Biofluid NfL concentrations have also been adopted as a response biomarker for evaluating candidate therapies in clinical trials for multiple neurological disorders, including HD. Evidence from trials in relapsing–remitting MS and progressive MS has shown that some therapies that effectively reduce axonal damage can lead to decreased NfL levels in both serum and CSF [66–71]. Studies in children with SMA type 1 treated with nusinersen, an ASO that modulates the splicing of the *SMN2* gene, showed a decrease in CSF NfL that was associated with improved motor function [46, 72]. Recently, a phase III trial in ALS patients with *SOD1* mutations showed that treatment with the SOD1-lowering ASO, tofersen, led to a reduction of plasma NfL but did not significantly improve clinical measures of disease [73]. Plasma NfL was accepted as a surrogate efficacy endpoint by the FDA in this trial based on the reasonable likelihood that its reduction would predict clinical benefit. This led to tofersen being granted accelerated approval for treatment of *SOD1* ALS.

CSF and/or blood NfL concentrations have been used as an exploratory endpoint of HD progression and therapeutic response in trials for HTT-lowering therapies [19–21, 74]. Preliminary results from the phase III GENERATION HD1 trial evaluating the efficacy of an ASO targeting the *HTT* transcript for degradation, tominersen (RO7234292), showed that CSF NfL concentrations were trending below baseline levels in response to sustained mHTT lowering in the CNS of individuals undergoing less frequent dosing (every 16 weeks) [20]. However, no improvements were seen overall in clinical or exploratory imaging outcome measures in response to tominersen treatment [20]. Additional studies evaluating the relationship between NfL levels in biofluids and mHTT lowering in the brain are needed for accurate interpretation of clinical NfL data.

In this study, we sought to investigate whether NfL concentrations in biofluids are responsive to allele-selective lowering of mHTT in the brain, and if the magnitude of mHTT lowering in the brain and/or the timing of intervention during the course of disease influences this response. We hypothesized that NfL concentrations in CSF and blood, which increase with disease progression in HD, would be stabilized or reduced in response to mHTT lowering in the brain. To test this, we utilized the well-characterized transgenic YAC128 mouse model of HD that expresses human, full-length mHTT and recapitulates many of the neuropathological, behavioural and biochemical features of the human disease [75]. YAC128 mice show reduced striatal volumes by MRI as early as 3 months [76, 77], motor deficits on the rotarod starting at 4 months [78–80], and selective striatal neuron loss by stereology from 9 months of age [75, 81], thus providing an opportunity to explore the relationship of mHTT lowering in the brain—initiated either prior to or after onset of HD-like motor deficits and neurodegeneration—with NfL concentrations in biofluids. We used single molecule array (SIMOA) for ultrasensitive measurement of NfL in cross-sectional CSF and longitudinal plasma samples from YAC128 and wild-type (WT) littermate control mice.

We first characterized the natural history of NfL in the CSF and plasma from YAC128 and WT littermate control mice up to 15 months of age. Next, we assessed CSF and plasma NfL concentrations over a 3-month interval in response to mHTT lowering in the CNS of YAC128 mice with either 15 µg (low-dose) or 50 µg (high-dose) of ASO targeting the human mutant *HTT* transcript (HTT ASO), initiated at either 2 or 12 months of age. Finally, we evaluated biofluid NfL concentrations over a 6-month interval in response to mHTT lowering in the CNS of YAC128 with high-dose HTT ASO, initiated at 8 months of age. These studies were designed to evaluate the response of NfL in biofluids to mHTT lowering in the brain initiated at different ages in YAC128 mice, with the aim of modeling therapeutic intervention during the premanifest (pre-symptomatic) and manifest (symptomatic) stages of HD.

## Methods

### Study design

The primary objectives of this study were to investigate whether NfL concentrations in plasma and CSF respond to mHTT lowering in the brain, and whether the magnitude and timing of this intervention may influence the response. We used the YAC128 mouse model of HD, which exhibits neuropathological, behavioural and biochemical features similar to those observed in people with HD. We characterized NfL concentrations in CSF and plasma over the natural history of disease in YAC128 and WT littermate control mice to assess whether YAC128 mice accurately model elevated NfL concentrations observed in HD mutation carriers. We evaluated NfL concentrations in biofluids following intraparenchymal injections of quinolinic acid (QA) into the striatum to elucidate NfL dynamics in CSF and plasma in response to acute neurotoxicity. Intracerebroventricular (ICV) injection of HTT ASO was used to lower mHTT in the CNS of YAC128 mice. Dosing was informed by previous studies using the same HTT ASO [25, 82], with the aim of achieving ~ 50% and ~ 75% mHTT reduction in the brain at a 1-month treatment interval with low- (15 µg) and high-dose (50 µg) HTT ASO treatment, respectively. Intervention timing was selected based on extensive phenotypic characterization of the YAC128 line. The early intervention time point (2 months of age) was selected to be an adult age prior to reported motor deficits and neuropathological phenotypes, to model therapeutic intervention during premanifest HD. The late intervention time point (12 months of age) was selected to be an age when YAC128 mice display robust regional forebrain atrophy and selective striatal neuron loss, to model intervention at a more advanced stage of disease. Finally, the mid intervention time point (8 months of age) was selected to be at an age when clear phenotypes in YAC128 are present but still progressing, in order to model intervention in an early/mid stage of HD. We evaluated CSF and plasma NfL concentrations over a 3-month interval in response to mHTT lowering in the CNS of YAC128 mice with either 15 µg or 50 µg HTT ASO, initiated at either 2 or 12 months of age. We then assessed biofluid NfL concentrations over a 6-month interval in response to mHTT lowering in the CNS of YAC128 with high-dose HTT ASO, initiated at 8 months of age.

### Mice

YAC128 mice and WT littermate controls were maintained under a 12-h light:dark cycle in a clean barrier facility and given ad libitum access to food and water. Approximately equal numbers of male and female mice were used for each experiment. Experiments were performed with approval from the Animal Care Committee at the University of British Columbia (A16-030 and A20-0107).

### CSF collection

Terminal CSF for cross-sectional NfL analysis was collected as previously described [25, 64]. Briefly, mice were anesthetized with Avertin (200 mg/kg, Sigma Aldrich, St. Louis, MO, catalog # T48402) by intraperitoneal (i.p.) injection and secured in a stereotaxic frame (Stoelting, Wood Dale, IL). The ear bars were raised and the nose piece used to position the mouse in a manner that would allow for a near 90° tilt of the head to access the cisterna magna. A 1 cm^2^ section of dorsal neck skin was removed, and muscle layers were dissected away to expose the dura, which was then cleaned and dried. A 50 µl Hamilton syringe with a 26-gauge (G) needle point style 2 (Hamilton, Reno, NV) was then lowered carefully into the cisterna magna. CSF was slowly withdrawn at a rate of 10 µl/min using an UltraMicroPump with Micro4 controller (World Precision Instruments, Sarasota, FL). CSF samples were centrifuged, then flash frozen in liquid nitrogen and stored at − 80 °C.

### Blood collection

Terminal blood collection for cross-sectional NfL analysis was performed via cardiac puncture where whole blood was drawn from the right ventricle using a 23 G needle and placed into prechilled EDTA-coated tubes (Sarstedt, Nümbrecht, Germany, catalog # 41.1395.105) on ice. Repeated blood sampling from the same animal for longitudinal NfL analysis was performed via the lateral saphenous vein where whole blood was drawn using EDTA-coated capillary blood collection tubes (Sarstedt, Nümbrecht, Germany, catalog # 16.444.100) and placed on ice. Whole blood was then centrifuged at 4000 relative centrifugal force (RCF) for 10 min at 4 °C, and plasma was carefully removed using a pipette, separated into aliquots, flash frozen in liquid nitrogen and stored at − 80 °C until use.

### CSF and plasma NfL analysis

NfL concentrations (pg/ml) were measured in mouse plasma and CSF using the commercially available NF-Light kit (Quanterix, Lexington, MA, catalog # 103186) on the SIMOA HD-1 analyzer (Quanterix). Plasma samples were diluted 1:40 and CSF samples were diluted 1:100 with NF-Light sample diluent provided within the kit. Then samples were loaded into the HD-1 analyzer and analyzed in duplicate as per the manufacturer’s instructions. The intra-assay coefficient of variance for each sample’s duplicate measurement was < 5%. All sample concentrations were above the lower limit of quantification for the assay (0.174 pg/ml).

### Tissue collection and processing

Mice allotted for terminal molecular and biochemical analyses were anesthetized using Avertin and CSF was collected as described above. Brains were then removed and placed on ice for 1 min to increase tissue rigidity, then microdissected into different regions (striatum, cortex, hippocampus, cerebellum). Samples were then flash frozen in liquid nitrogen and stored at − 80 °C until use.

Mice allotted for histology were perfused transcardially with phosphate-buffered saline (PBS) for 4 min then with 4% paraformaldehyde (PFA) in PBS for 3 min. Brains were removed and post-fixed in 4% PFA for 24 h at 4 °C. The following day, brains were cryoprotected in 30% sucrose in PBS with 0.01% sodium azide. Once equilibrated, the brains were divided into forebrain and cerebellum, and forebrains were frozen on dry ice, mounted in Tissue-TEK O.C.T. compound (Sakura, catalog # 4583), and cut using the Leica CM3050 S cryostat (Leica, Wetzlar, Germany) into a series of 25-μm coronal sections free-floating in PBS with 0.01% sodium azide.

### Quantitative polymerase chain reaction (qPCR)

Striatum samples were lysed using MP Biomedicals Lysing Matrix D tubes (Thermo Fisher Scientific, Waltham, MA, catalog # MP116913500) and the FastPrep FP120 Cell Disrupter (Thermo Electron, Waltham, MA). RNA extraction was performed using the RNeasy Mini Kit (Qiagen, Hilden, Germany, catalog # 74106) according to the manufacturer’s protocol. cDNA synthesis was performed using the SuperScript III First-Strand Synthesis System (Invitrogen, Waltham, MA, catalog # 18080051) according to the manufacturer’s protocol using 250 ng or 1000 ng of RNA for striatum and cortex, respectively.

For qPCR, primers for mouse *Nefl* (forward 5′ GGG TAT GAA CGA AGC TCT GG-3′; reverse 5′-TCT CAG CTC ATT CTC CAG TTT G 3′), *Gfap* (forward 5′ GAA AAC CGC ATC ACC ATT CC 3′; reverse 5′ CTT AAT GAC CTC ACC ATC CCG 3′) and *Rpl13a* (forward 5′-GGA GGA GAA ACG GAA GGA AAA G-3′; reverse 5′-CCG TAA CCT CAA GAT CTG CTT CTT-3′) were synthesized by Integrated DNA Technologies (IDT, Newark, NJ). cDNA was diluted 1:3 for the striatum or 1:5 for the cortex in nuclease-free dH_2_O. SYBR Green PCR Master Mix (Thermo Fisher Scientific, Waltham, MA, catalog # 4368706) reagents were used according to the manufacturer’s protocol and qPCR was run using the Applied Biosystems 7500 Fast Real-Time PCR System (Thermo Fisher Scientific). Relative quantitation of transcript levels was performed using the 2^−ΔΔCT^ method.

### QA injection

Unilateral intrastriatal QA injections were performed as previously described [64, 83]. Briefly, mice were anesthetized with ketamine (150 mg/kg) and xylazine (10 mg/kg) by i.p. injection and secured into a stereotaxic frame. The scalp was shaved, sterilized with betaine and 70% ethanol, and an incision made along the midline. The skull was dried to enhance visibility of sutures and landmarks. A dental drill was used to make a burr hole on the right side of the skull at 0.8 mm anterior and 1.8 mm lateral to bregma. A Hamilton syringe with a 26 G needle point style 2 (Hamilton, Reno, NV) loaded with either 2 µl of PBS or QA (25 nM in sterile PBS) was lowered to a depth of 3.5 mm through the burr hole, and injected at 0.5 µl/min using an UltraMicroPump with Micro4 controller (World Precision Instruments, Sarasota, FL). The needle was left in place for 5 min and then withdrawn slowly. Plasma was collected prior to treatment and at 3 and 7 days post-treatment whereas brains and CSF samples were collected at 3 or 7 days post-treatment.

### Histology

For detection of degenerating neurons after QA treatment, sections spanning the injection site were stained using Fluoro-Jade C (Biosensis, Thebarton, South Australia, catalog #TR-100-FJ) according to the manufacturer's protocol. Sections were mounted using ProLong Gold Antifade mountant with DAPI (Invitrogen, catalog # P36941), and imaged with a 2.5 × objective using a Zeiss Axioplan 2 microscope (Carl Zeiss AG, Oberkochen, Germany).

For visualization of mHTT inclusions in the brain, sections were co-stained with mouse EM48 (1:250, Sigma-Aldrich, Burlington, MA, catalog # MAB5374) and chicken NeuN (1:500, Sigma-Aldrich, catalog # ABN91). Primary antibodies were detected with goat anti-mouse AlexaFluor-488 (1:1000, Invitrogen, catalog # A-11029) and goat anti-chicken AlexaFluor-647 (1:500, Invitrogen, catalog # A-21449) secondary antibodies. Sections were then mounted using ProLong Gold Antifade mountant with DAPI (Thermo Fisher Scientific, catalog # P36931) and imaged with a 40 × objective using a Leica TCS SP5 laser scanning confocal microscope.

### HTT ASO design

A 5–9-5 chimeric gapmer ASO with a fully phosphorothioate-modified backbone and 5 locked-nucleic acid modifications at each wing designed to target intron 22 of the human *HTT* transcript was synthesized by Qiagen (Hilden, Germany) [25]. For ASO treatments, molecules were diluted to the indicated concentrations in sterile PBS and administered by ICV injection as described below.

### ICV injections

Unilateral ICV injections were performed as previously described [64]. Briefly, mice were anesthetized with isoflurane and secured into a stereotaxic frame (Stoelting). The scalp was prepared as described above. A Hamilton syringe with a 26 G needle point style 2 (Hamilton, Reno, NV) loaded with either 10 µl of PBS or 50 µg HTT ASO diluted in PBS was punched through the skull at − 1 mm lateral, + 0.3 mm anterior and − 3.0 mm ventral to bregma. PBS and HTT ASO were delivered as a bolus. The needle was left in place for 2 min after injection and withdrawn slowly to limit backflow.

### Quantitative immunoblotting

Tissues were lysed through mechanical dissociation and triturated in lysis buffer (50 mM Tris pH 8.0, 150 mM NaCl, 1% NP40, 40 mM β-glycerophosphate, 10 mM NaF) containing protease inhibitors (1 mM Roche Complete mini EDTA free, 1 nM NaVan, 1 mM PMSF, and 5 µM zVAD). Lysates were incubated on ice and vortexed every 5 min for 20 min. Lysates were centrifuged for 15 min at 18,213 RCF at 4 °C and the supernatant was collected. Total protein was quantified using the DC protein assay (Bio-Rad, Hercules, CA, catalog # 5000113 and 5000114). Samples containing 50 µg total protein were prepared using NuPAGE LDS Sample Buffer (Invitrogen, catalog # NP0007) containing 50 mM dithiothreitol and denatured at 70 °C for 10 min.

For quantitation of NfL levels in the brain, 75 μg of protein was resolved using 12% bis–Tris gels run in SDS-Tris–Glycine running buffer (1 × concentration: 25 mM Tris base, 190 mM glycine, and 0.1% SDS). Gels were transferred to 0.2-μm nitrocellulose membranes for 2 h at 18 V in Tris–Glycine transfer buffer (25 mM Tris Base, 190 mM glycine, and 5% MeOH). Membranes were blocked for 30 min in 5% skim milk in PBS with 0.5% Tween-20 (PBS-T) at room temperature with shaking followed by 4 × 5 min washes with PBS-T. Membranes were incubated with rabbit NfL (1:1000, Cell Signaling Technology, Danvers, MA, catalog # 2837) or mouse actin (1:5000, Sigma-Aldrich, catalog # MAB1501R) primary antibody diluted in 5% BSA in PBS-T with 0.01% NaN_3_ overnight at 4 °C. Membranes were then washed in PBS-T for 3 × 10 min and incubated with IRDye 800CW goat anti-rabbit (1:5000, LI-COR Biosciences, Lincoln, NE, catalog # 926–3221) or Alexa Fluor 680 goat anti-mouse (1:5000, Invitrogen, catalog # A21058) secondary antibody diluted in 5% skim milk in PBS-T for 1 h, protected from light. Finally, membranes were washed 4 × 5 min with PBS-T and imaged using the LI-COR Odyssey Infrared Imaging System (LI-COR Biosciences). Densitometry of NfL and actin bands was performed using the LI-COR Image Studio Lite software, and NfL intensities were normalized to actin loading control.

For quantitation of mHTT levels in the brain, HTT alleles were resolved as previously described [84]. Briefly, 50 μg of total protein was resolved using 10% low bis:acrylamide (1:200) gels run in SDS-Tris–glycine running buffer (1 × buffer concentration: 25 mM Tris base, 190 mM glycine, 3.5 mM SDS and 10.7 mM β-mercaptoethanol). Gels were transferred for 2 h onto a 0.45-μm nitrocellulose membrane at 18 V in NuPage transfer buffer (1 × buffer concentration: 25 mM Bicine, 25 mM Bis–Tris, 1 mM EDTA, and 5% methanol). Membranes were then blocked with 5% skim milk in PBS-T for 30 min at room temperature with shaking followed by 4 × 5 min washes with PBS-T. Blots were then probed with mouse 1CU-4C8 (Thermo Fisher Scientific, catalog # MAB2166) or rabbit calnexin (Sigma-Aldrich, catalog # C4731) primary antibody diluted in 5% BSA in PBS-T with 0.01% NaN_3_ overnight at 4 °C. Membranes were then washed in PBS-T for 3 × 10 min and incubated with IR dye 800CW goat anti-mouse (1:5000, Rockland Immunochemicals, Philadelphia, PA, catalog # 610–131-007) or AlexaFluor 680 goat anti-rabbit (1:5000, Invitrogen, catalog # A21076) secondary antibody diluted in 5% skim milk in PBS-T for 1 h, protected from light. Finally, membranes were washed 4 × 5 min with PBS-T and imaged using the LI-COR Odyssey Infrared Imaging system (LI-COR Biosciences). Densitometry on HTT and calnexin bands was performed using the LI-COR Image Studio Lite software, and background-subtracted HTT intensities were normalized to calnexin loading control and then to the same allele of vehicle-treated mice on the same membrane.

### Mesoscale Discovery (MSD) aggregate analysis

Quantification of mHTT aggregates in cortical samples from YAC128 mice was performed at Evotec (Hamburg, Germany) by the MSD assay using HTT antibodies MW8 (Developmental Studies Hybridoma Bank, Iowa City, IA, catalog # MW8) and 4C9-sulfo as previously described [85].

### Statistical analysis

Statistical analyses were performed using GraphPad Prism version 10 (GraphPad) and R statistical software version 4.3.0 [86]. Statistical tests used are described in the figure legends. *P* < 0.05 was considered as statistically significant for all analyses.

NfL concentrations in CSF and plasma were assessed for normality and found to be non-normally distributed. Natural log transformation was found to produce a normal distribution, as reported previously in human HD CSF [27–29]. Transformed values were used for all analyses, except in the presentation of the trajectories of CSF and plasma NfL over the natural history of disease in YAC128 and WT littermate control mice, where untransformed NfL concentrations were used.

Comparisons between genotypes and/or treatment groups were performed using either a two-tailed Student’s *t*-test (2 groups) or ANOVA (more than 2 groups). Comparisons of longitudinal NfL data over the natural history involving repeated measures of samples from the same animal were performed by mixed-effects models. *Post-hoc* analyses were performed using the Tukey test (one-way or two-way ANOVA) or the Šidák test (two-way ANOVA) to correct for multiple comparisons. Longitudinal plasma NfL concentrations in response to mHTT lowering were compared over time by treatment group using linear mixed effects models with a random intercept for each animal. To allow for possible nonlinearity over time, we fit time with a 3 degree of freedom natural spline. To allow for differences in shape in each treatment group, we included an interaction term between these splines and treatment. Models were adjusted for baseline NfL concentrations. Results are summarized graphically as fitted values over time (by treatment group) where solid lines denote the mean and shaded bands denote 95% confidence intervals (95% CI) for each treatment condition, as well as in tables as estimated mean differences (MD) over time between treatment groups. The latter were calculated at the average baseline value, and were computed at each time point data where available. We also present smoothed curves over time including baseline values where solid lines denote the mean and shaded bands denote 95% CI for each treatment condition. Models were fitted using the lmer function in the Ime4 package in R [87].

Receiver operating characteristic (ROC) curve analysis was used to compare the discriminatory performance of CSF and plasma NfL for distinguishing between genotypes. Simple linear regression was used to evaluate the relationship of HTT ASO dose with CSF NfL concentrations. Correlation analyses were performed using Pearson’s correlation, where coefficient values (Pearson’s *r*) from ± 0.50 to ± 1 were considered strong associations, ± 0.30 to ± 0.49 were considered moderate associations, and ± 0.10 to ± 0.29 were considered weak associations.

Box and whisker plots were used to show distribution and skewness of data. Boxes show the interquartile range (IQR), whiskers extend from minimum to maximum values, horizontal lines show the median, and crosses show the mean.

## Results

### Plasma and CSF NfL concentrations are elevated over the natural history of disease in YAC128 mice

**Fig. 1:**
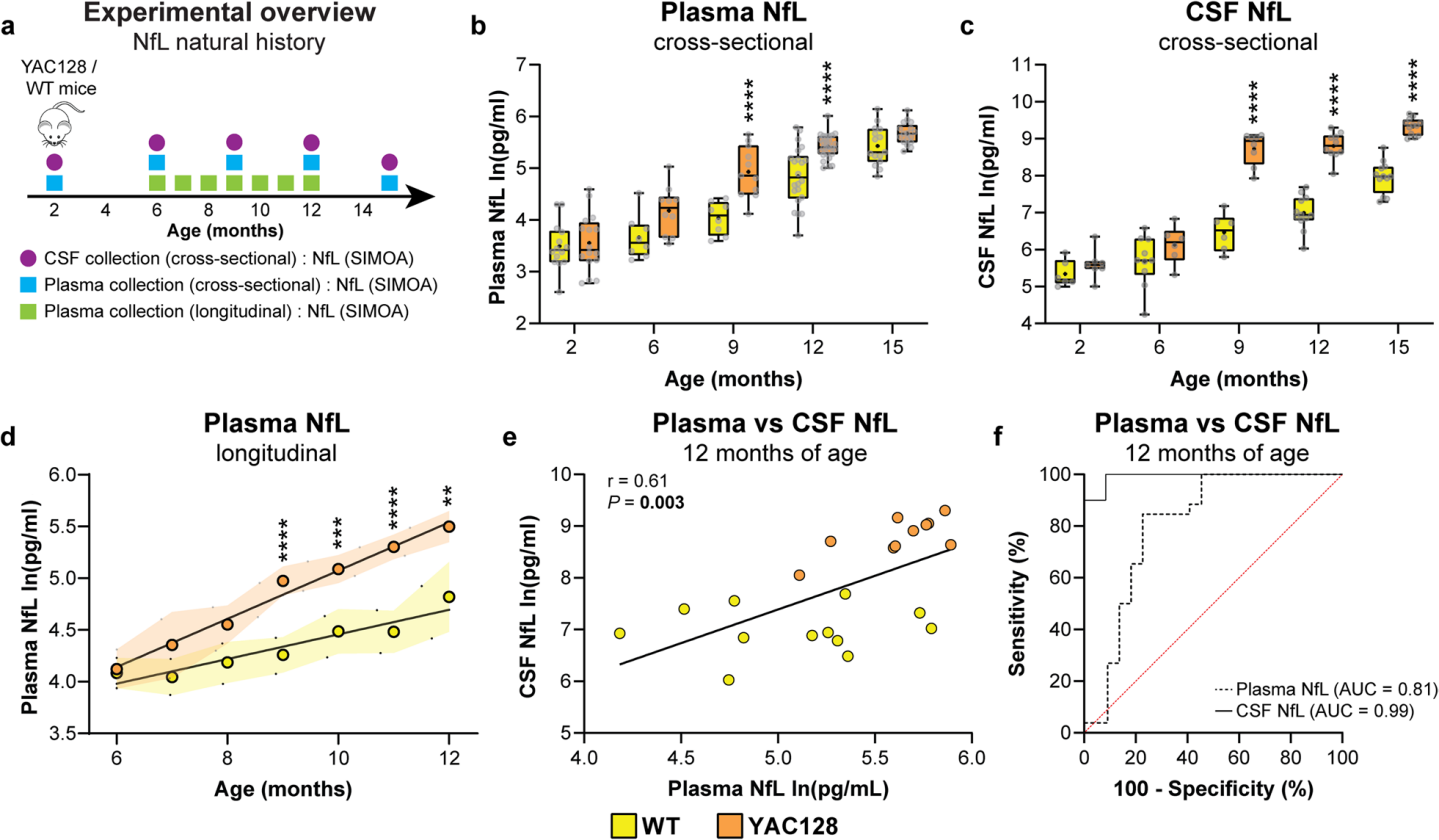
Plasma and CSF NfL concentrations are elevated over the natural history of disease in YAC128 mice. **a** Schematic diagram of experimental design. **b**, **c** Cross-sectional NfL concentrations (natural log pg/ml) in plasma (2 months: *n* = 14 WT, *n* = 16 YAC128; 6 months: *n* = 8 WT, *n* = 10 YAC128; 9 months: *n* = 8 WT, *n* = 11 YAC128; 12 months: *n* = 22 WT, *n* = 26 YAC128; 15 months: *n* = 15 WT, *n* = 17 YAC128; two-way ANOVA: genotype *P* < 0.0001, age *P* < 0.0001, interaction *P* = 0.008; ****P < 0.0001) and CSF from YAC128 and WT littermate control mice (2 months: *n* = 6 WT, *n* = 7 YAC128; 6 months: *n* = 10 WT, *n* = 6 YAC128; 9 months: *n* = 6 WT, *n* = 8 YAC128; 12 months: *n* = 12 WT, *n* = 10 YAC128; 15 months: *n* = 12 WT, *n* = 12 YAC128; two-way ANOVA: genotype *P* < 0.0001, age *P* < 0.0001, interaction *P* < 0.0001; ****P < 0.0001). Boxes show the IQR, whiskers extend from the minimum to maximum value, horizontal lines show the median, and crosses show the mean. **d** Longitudinal plasma NfL concentrations from 6 to 12 months of age in WT and YAC128 mice (WT: *n* = 21, YAC128: *n* = 19; mixed-effect analysis: genotype *P* < 0.0001, age *P* < 0.0001, genotype × age *P* < 0.0001; **P = 0.005, ***P = 0.0002, ****P < 0.0001). A linear trend line was fitted to mean values for each genotype and shaded bands denote the 95% CI. **e** Correlation between NfL concentrations from paired plasma and CSF samples in WT and YAC128 mice at 12 months of age (WT: *n* = 12, YAC128: *n* = 10; Pearson’s correlation: *r* = 0.61, *P* = 0.003). **f** ROC curve comparing sensitivity and specificity of plasma NfL (WT: *n* = 12, YAC128: *n* = 10. AUC = 0.81, *P* = 0.0003) and CSF NfL (AUC = 0.99, *P* = 0.0001) for discriminating between YAC128 and WT mice at 12 months of age. Plasma and CSF NfL concentrations are natural log transformed

The natural history of NfL in biofluids was characterized in the YAC128 mouse model of HD. Cross-sectional CSF and plasma were collected from YAC128 and WT littermate control mice at 2, 6, 9, 12 and 15 months of age for measurement of NfL concentrations (Fig. 1a). In a replication cohort, longitudinal plasma samples were collected at monthly intervals from 6 to 12 months of age for measurement of NfL concentrations (Fig. 1a).

Cross-sectional plasma NfL concentrations were significantly elevated in YAC128 compared to WT mice at 9 and 12 months, but not at 15 months of age (Fig. S1a, untransformed NfL concentrations; Fig. 1b, natural log transformed NfL concentrations). We also detected a significant increase of CSF NfL concentration in YAC128 compared to WT mice from 9 months of age (Fig. S1b, untransformed NfL concentrations; Fig. 1c, natural log transformed NfL concentrations).

Consistent with cross-sectional data, longitudinal plasma NfL concentrations were significantly elevated in YAC128 compared to WT mice from 9 months of age (Fig. S1c, untransformed NfL concentrations; Fig. 1d, natural log transformed NfL concentrations).

A strong positive correlation between paired plasma and CSF NfL concentrations was found in both genotypes at 12 months of age (Fig. 1e). ROC curve analysis was performed to compare the sensitivity and specificity of plasma and CSF NfL for stratifying YAC128 from WT mice at 12 months of age (Fig. 1f). Both biofluids showed strong discrimination between genotypes, with CSF NfL showing superior accuracy compared to plasma NfL at this age.

### Acute neurodegeneration in the striatum of YAC128 mice leads to increased plasma and CSF NfL concentrations

**Fig. 2:**
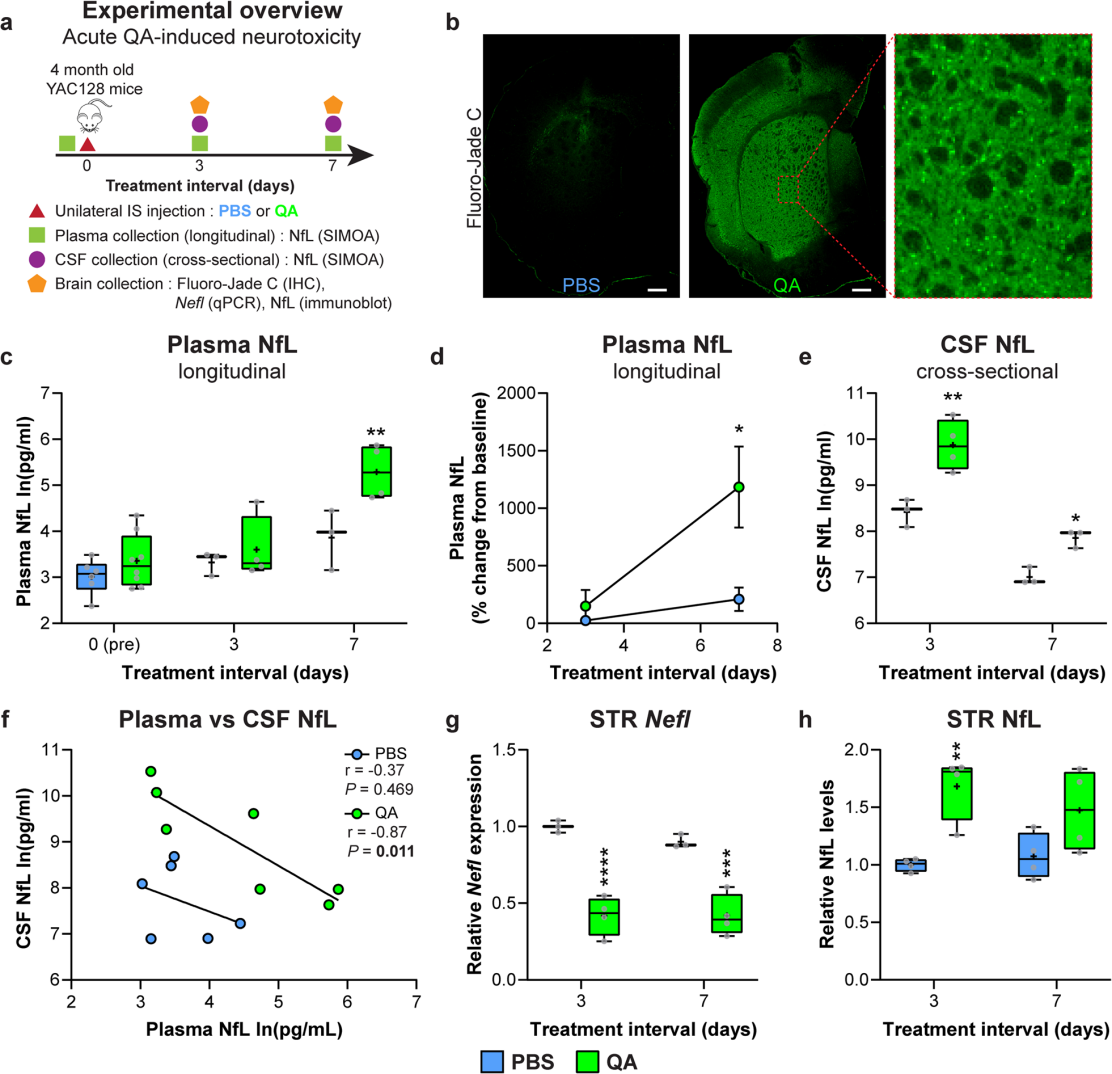
Acute neurodegeneration in the striatum of YAC128 mice leads to increased plasma and CSF NfL concentrations. **a** Schematic diagram of the experimental design. **b** IHC images of Fluoro-Jade C staining in YAC128 mice injected with either PBS (left) or QA (middle) on day 3 after injection. Scale bar, 500 µm. Inset panel shows higher-magnification view of the QA-injected striatum. **c** Longitudinal plasma NfL concentrations at baseline and on days 3 and 7 after injection with PBS or QA (baseline: *n* = 6 PBS, *n* = 8 QA; 3 days: *n* = 3 PBS, *n* = 4 QA; 7 days: *n* = 3 PBS, *n* = 4 QA; two-way ANOVA: treatment *P* = 0.005, interval *P* < 0.0001, interaction *P* = 0.093; **P = 0.007). Boxes show the IQR, whiskers extend from minimum to maximum values, horizontal lines show the median, and crosses show the mean. **d** Percent change of plasma NfL concentrations from baseline for each animal at 3 and 7 days post-injection with PBS or QA (two-way ANOVA: treatment *P* = 0.037, interval *P* = 0.024, interaction *P* = 0.093; *P = 0.026). Symbols show the mean and error bars represent SEM. **e** Cross-sectional CSF concentrations on days 3 and 7 after injection with PBS or QA (3 days: *n* = 3 PBS, *n* = 4 QA; 7 days: *n* = 3 PBS, *n* = 3 QA; two-way ANOVA: treatment *P* = 0.0003, interval *P* < 0.0001, interaction *P* = 0.176; *P = 0.040, **P = 0.001). **f** Correlation between NfL concentrations from paired plasma and CSF samples on days 3 and 7 post-injection with PBS (Pearson’s correlation: *r* =  − 0.37, *P* = 0.469) or QA (Pearson’s correlation:* r* =  − 0.87, *P* = 0.011). **g**, **h** Relative *Nefl* expression (3 days: *n* = 3 PBS, *n* = 4 QA; 7 days: *n* = 3 PBS, *n* = 4 QA; two-way ANOVA: treatment *P* < 0.0001, interval *P* = 0.403, interaction *P* = 0.394; ***P = 0.0003, ****P < 0.0001) and NfL protein level (3 days: *n* = 4 PBS, *n* = 4 QA; 7 days: *n* = 4 PBS, *n* = 4 QA. Two-way ANOVA: treatment *P* = 0.001, interval *P* = 0.603, interaction *P* = 0.279. **P = 0.005) in the injected striatum following unilateral intrastriatal injection of PBS or QA. Fold changes are presented relative to PBS values at day 3 post-QA injection. Plasma and CSF NfL concentrations are natural log transformed

We next assessed the dynamics of biofluid NfL in response to acute neurodegeneration in the brain. QA, a neurotoxin known to induce excitotoxicity [83, 88], was administered unilaterally into the striatum of YAC128 mice at 4 months of age. NfL concentrations were measured in longitudinal plasma and cross-sectional CSF samples on days 3 and 7 after PBS or QA injection (Fig. 2a). Brains were also collected to measure *Nefl* transcript and NfL protein levels in the injected striatal hemisphere.

The presence of a striatal lesion following QA administration was confirmed by immunohistochemistry using the Fluoro-Jade C dye, which stains degenerating neurons (Fig. 2b). Plasma NfL concentrations were significantly elevated with QA compared to PBS on day 7, but not on day 3 after injection (Fig. 2c). Notably, administration of QA resulted in a ~ 149% increase of plasma NfL from baseline on day 3, and > 1000% increase from baseline on day 7 after injection (Fig. 2d).

CSF NfL concentrations in animals injected with either PBS or QA were both higher on day 3 than on day 7 after injection, with NfL levels being significantly increased at both time points in animals that received QA (Fig. 2e). A moderate negative correlation of NfL concentrations was observed between paired CSF and plasma samples in animals injected with PBS at both time points, whereas there was a strong negative correlation with QA (Fig. 2f).

*Nefl* transcript levels were significantly reduced in the striatum with QA injection at both time points (Fig. 2g). Conversely, NfL protein levels were significantly increased in the injected striatum on day 3, with a trend towards an increase on day 7 (*P* = 0.085) following QA injection (Fig. 2h).

### Sustained, dose-dependent lowering of mHTT in the brains of YAC128 mice with HTT ASO

We performed a time-course experiment to characterize the magnitude and duration of mHTT lowering in the brains of YAC128 mice following ICV administration of two doses of HTT ASO. Dose selection was informed by previous studies using the same HTT ASO [25, 82], with the aim of achieving either 40%–50% or 70%–85% mHTT reduction in the brain at a 1-month treatment interval with low- or high-dose HTT ASO treatment, respectively. We therefore selected 15 µg HTT ASO and 50 µg HTT ASO for all subsequent experiments.

**Fig. 3:**
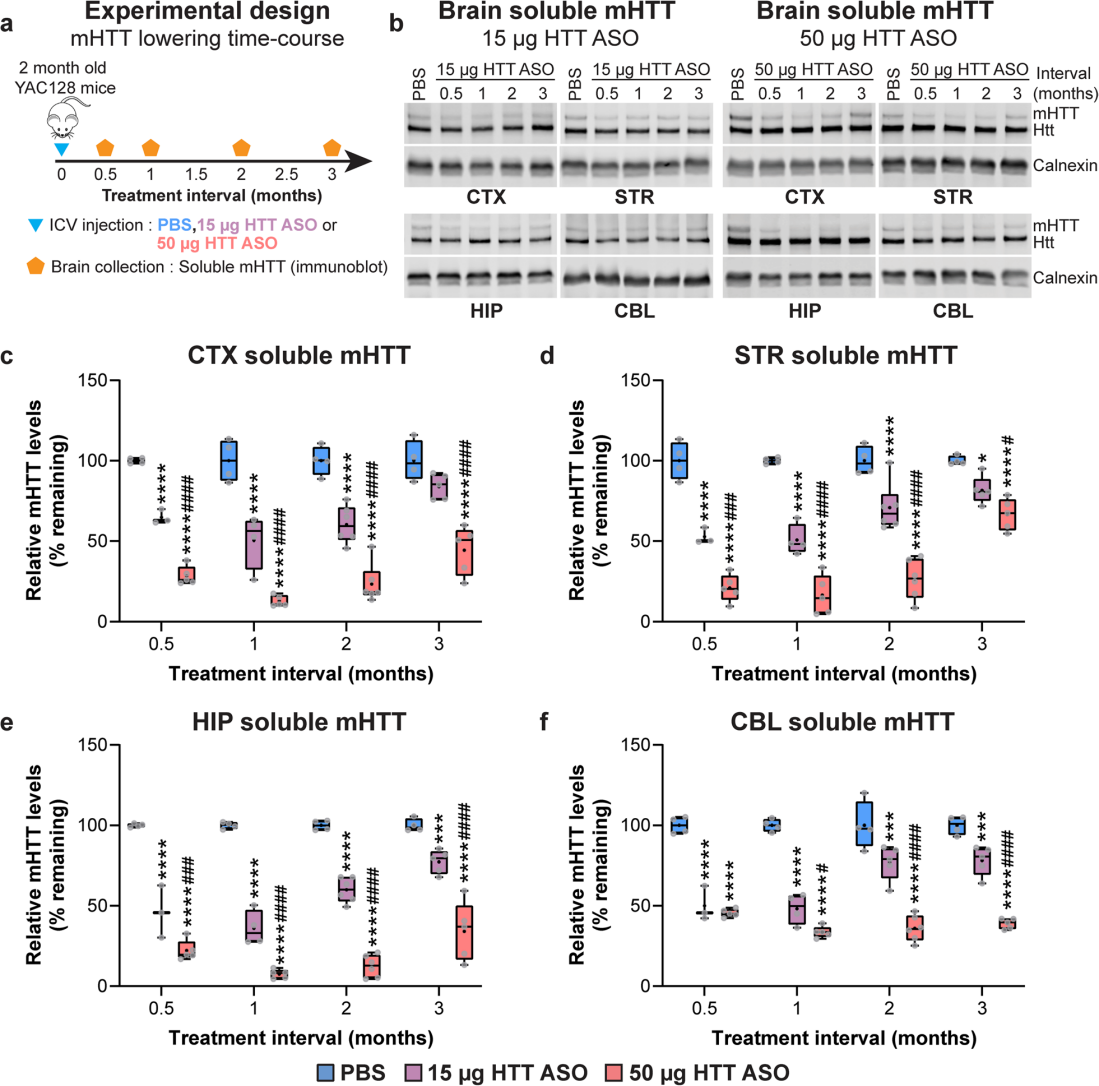
Sustained, dose-dependent lowering of mHTT in the brains of YAC128 mice with HTT ASO treatment. **a** Schematic diagram of experimental design (0.5-month interval: *n* = 4 PBS, *n* = 3 15 µg HTT ASO, *n* = 5 50 µg HTT ASO; 1-month interval: *n* = 4 PBS, *n* = 4 15 µg HTT ASO, *n* = 5 50 µg HTT ASO; 2-month interval: *n* = 4 PBS, *n* = 6 15 µg HTT ASO, *n* = 6 50 µg HTT ASO; 3-month interval: *n* = 4 PBS, *n* = 5 15 µg HTT ASO, *n* = 5 50 µg HTT ASO). **b** Representative immunoblots from brain lysates of YAC128 mice treated at 2 months of age with either PBS, 15 µg HTT ASO (left panel) or 50 µg HTT ASO (right panel), collected at intervals up to 3 months post-treatment. **c**–**f** Quantification of soluble mHTT levels in the (**c**) cortex (Two-way ANOVA: treatment *P* < 0.0001, interval *P* < 0.0001, interaction *P* = 0.0319; ****P < 0.0001 vs PBS; ^####^*P* < 0.0001 vs 15 µg HTT ASO), (**d**) striatum (Two-way ANOVA: treatment *P* < 0.0001, interval *P* < 0.0001, interaction *P* < 0.0001; *P = 0.018, ****P < 0.0001 vs PBS; ^#^*P* = 0.046, ^###^*P* = 0.0001, ^####^*P* < 0.0001 vs 15 µg HTT ASO), (**e**) hippocampus (two-way ANOVA: treatment *P* < 0.0001, interval *P* < 0.0001, interaction *P* < 0.0001; ***P = 0.0005, ****P < 0.0001 vs PBS; ^###^*P* = 0.0007, ^####^*P* < 0.0001 vs 15 µg HTT ASO), and (**f**) cerebellum (two-way ANOVA: treatment *P* < 0.0001, interval *P* = 0.0006, interaction *P* < 0.0001; ***P = 0.0001, ****P < 0.0001 vs PBS; ^#^*P* = 0.017, ^####^*P* < 0.0001 vs 15 µg HTT ASO). Levels of mHTT in all brain regions are normalized to PBS values at each respective time point. Boxes show the IQR, whiskers extend from minimum to maximum values, horizontal lines show the median, and crosses show the mean

Two-month-old YAC128 mice received a single unilateral ICV injection with PBS, 15 µg HTT ASO or 50 µg HTT ASO. Levels of mHTT were measured by quantitative immunoblotting [84] in different brain regions (cortex, striatum, hippocampus and cerebellum) at 0.5-, 1-, 2- and 3-month post-treatment intervals (Fig. 3a, b). The HTT ASO used in this study targets the human mutant *HTT* transgene in YAC128 mice without altering the level of mouse Htt [82]. For all subsequent experiments, we report levels of mHTT protein in the brain as a measure of target engagement.

We observed a significant interaction between treatment condition and interval in all brain regions, with both doses of HTT ASO resulting in a significant reduction of mHTT compared to PBS in the cortex (Fig. 3c), striatum (Fig. 3d), hippocampus (Fig. 3e) and cerebellum (Fig. 3f).

As expected, we observed a greater magnitude of mHTT lowering with 50 µg compared to 15 µg HTT ASO in each brain region at all time points assessed (Fig. 3c–f). For both doses of HTT ASO, the highest magnitude of mHTT lowering in all brain regions was observed at approximately 1 month post-treatment (15 µg HTT ASO in cortex: MD =  − 49.51%, striatum: MD =  − 49.29%, hippocampus: MD =  − 64.03%, cerebellum: MD =  − 51.85%; 50 µg HTT ASO in cortex: MD =  − 87.11%, striatum: MD =  − 83.49%, hippocampus: MD =  − 92.51%, cerebellum: MD =  − 66.38%; Fig. 3c–f).

At 3 months after treatment, mHTT levels in the 15 µg HTT ASO-treated animals were trending back towards baseline (cortex: MD =  − 16.00%, striatum: MD =  − 18.38%, hippocampus: MD =  − 22.64%, cerebellum: MD =  − 21.91%), whereas more sustained mHTT reduction was observed with 50 µg HTT ASO in all brain regions up to this time point (cortex: MD =  − 55.69%, striatum: MD =  − 33.40%, hippocampus: MD =  − 65.97%, cerebellum: MD =  − 61.05%; Fig. 3c–f).

We extended our time-course with 50 µg HTT ASO to include measurement of mHTT at 4-, 5- and 6-month post-treatment intervals (Fig. S2a). Levels of mHTT in the cortex were not significantly decreased with 50 µg HTT ASO at the three intervals (Fig. S2b). In contrast, mHTT was significantly reduced in the striatum and the hippocampus at the 4-month interval with 50 µg HTT ASO, and returned to near baseline levels at the 5-month interval (Fig. S2c, d). In the cerebellum, mHTT was significantly decreased at the 4- and 5 month interval, and returned to near baseline levels at the 6-month post-treatment interval (Fig. S2e). For subsequent studies, 3- and 6-month treatment paradigms were described as short- and long-duration mHTT lowering, respectively.

### Lowering mHTT in the brains of YAC128 mice prior to motor deficits and regional forebrain atrophy leads to a reduction of CSF NfL concentration

**Fig. 4:**
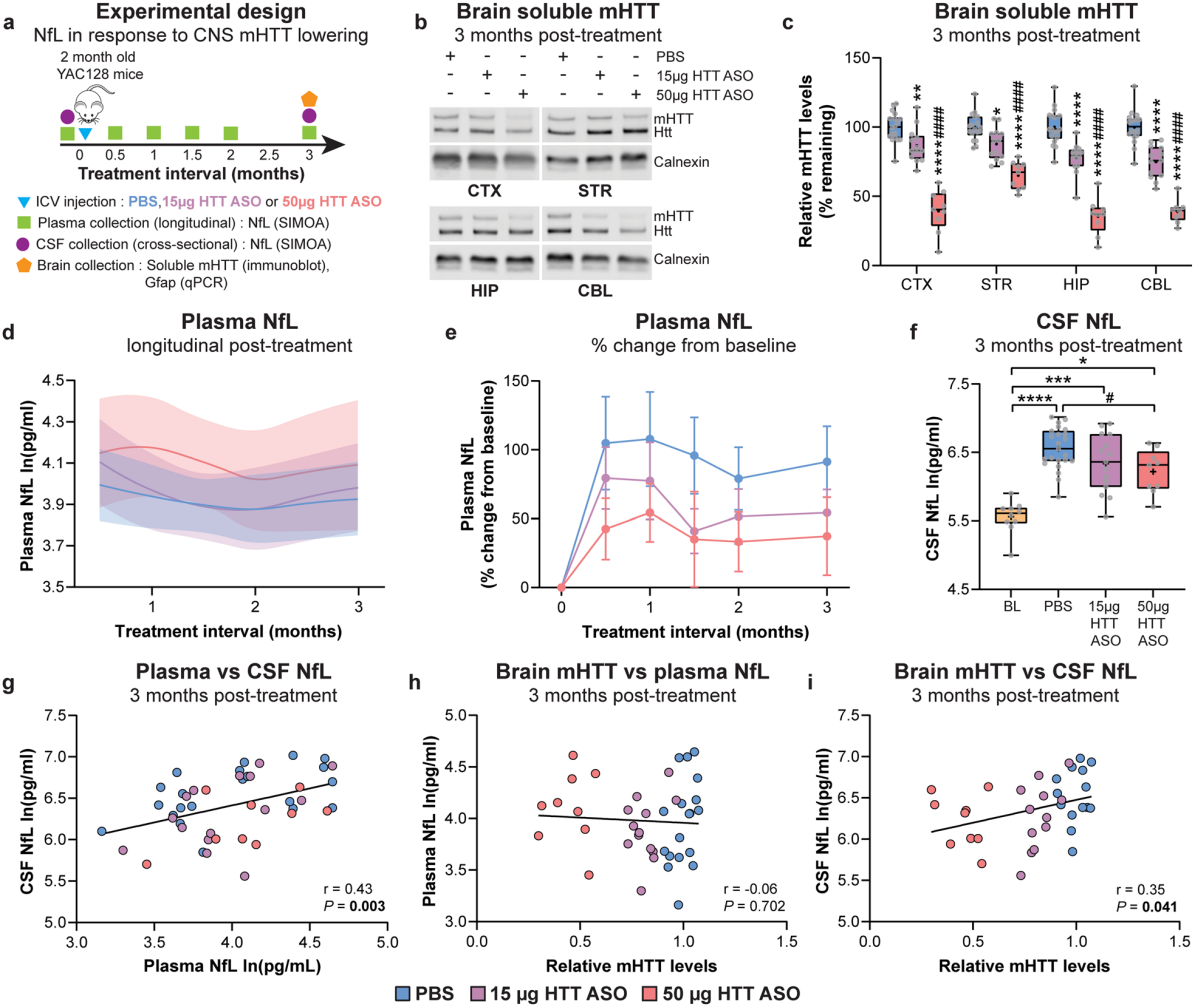
Lowering mHTT in the brains of YAC128 mice prior to motor deficits and regional forebrain atrophy leads to a reduction of CSF NfL concentration. **a** Schematic diagram of the experimental design. **b**, **c** Representative immunoblots and quantification of soluble mHTT levels in the CTX, STR, HIP and CBL of YAC128 mice treated at 2 months of age with either PBS, 15 µg HTT ASO or 50 µg HTT ASO, and assessed 3 months post-treatment (PBS: *n* = 17; 15 µg HTT ASO: *n* = 10; 50 µg HTT ASO: *n* = 12; two-way ANOVA: treatment *P* < 0.0001, brain region *P* < 0.0001, interaction *P* = 0.0001; *P = 0.013, **P = 0.006, ****P < 0.0001 vs PBS; ^###^*P* = 0.0007, ^####^*P* < 0.0001 vs 15 µg HTT ASO). Levels of mHTT are normalized to PBS values. Boxes show the IQR, whiskers extend from minimum to maximum values, horizontal lines show the median, and crosses show the mean. **d** Longitudinal plasma NfL concentrations following treatment with PBS, 15 µg HTT ASO or 50 µg HTT ASO (PBS: *n* = 29; 15 µg HTT ASO: *n* = 20; 50 µg HTT ASO: *n* = 13; linear mixed-effects model: treatment *P* = 0.151, treatment × time *P* = 0.983). Models were adjusted for time from treatment and baseline NfL concentrations for each treatment condition, and fitted with a cubic spline. Solid lines denote the mean and shaded bands denote the 95% CI for each treatment condition. **e** Percent change in longitudinal plasma NfL concentrations from baseline for each animal at multiple time points (mixed-effects model: treatment *P* = 0.190, time *P* = 0.396, treatment × time *P* = 0.998). Symbols show the mean and error bars represent SEM. **f** Cross-sectional CSF NfL concentrations in YAC128 mice at 2 months of age (baseline, BL) and 3 months post-treatment with either PBS, 15 µg HTT ASO or 50 µg HTT ASO (BL: *n* = 10; PBS: *n* = 23; 15 µg HTT ASO: *n* = 15; 50 µg HTT ASO: *n* = 9; one-way ANOVA: *P* < 0.0001; *P = 0.029, ***P = 0.0004, ****P < 0.0001; ^#^*P* = 0.043. Linear regression: Y = -0.0068X + 6.520,* R*^2^ = 0.12, *P* = 0.0161). **g** Correlation between NfL concentrations from paired plasma and CSF samples (Pearson’s correlation: *r* = 0.43, *P* = 0.003). **h, i** Correlations between whole-brain mHTT level (mean mHTT from all brain regions for each animal) and (**h**) plasma (Pearson’s correlation:* r* = -0.06, *P* = 0.702) or (**i**) CSF NfL (Pearson’s correlation: *r* = 0.35, *P* = 0.041) from the same animal at 3 months post-treatment with PBS, 15 µg HTT ASO or 50 µg HTT ASO. Plasma and CSF NfL concentrations are natural log transformed

We evaluated longitudinal plasma NfL and cross-sectional CSF NfL over a 3-month interval in response to low- or high-dose HTT ASO treatment initiated at 2 months of age (Fig. 4a). Brains were collected at 3 months post-treatment to measure levels of full-length, soluble mHTT protein and *Gfap* mRNA expression in the cortex, striatum, hippocampus and cerebellum.

A significant interaction between treatment condition and brain region was observed on mHTT level at the 3-month treatment interval (Fig. 4b, c). Full-length, soluble mHTT was significantly reduced in the cortex, striatum, hippocampus and cerebellum following treatment with 15 µg HTT ASO and 50 µg HTT ASO at the 3-month post-treatment interval. *Gfap* expression was not altered following treatment with 50 µg HTT ASO compared to PBS in any brain region assessed (Fig. S3).

Longitudinal concentrations of plasma NfL were not significantly changed in response to treatment with either 15 or 50 µg HTT ASO compared to PBS over a 3-month interval (Fig. S4, model including baseline NfL concentrations; Fig. 4d, model adjusted for baseline NfL concentrations). Treatment with 15 µg HTT ASO showed little effect on plasma NfL concentrations at any time point over the 3-month interval (Table 1). YAC128 mice treated with 50 µg HTT ASO showed a trend toward increased plasma NfL compared to PBS at each time point over the 3-month interval, which was highest at 1-month post-treatment (*P* = 0.098). Plasma NfL concentrations did not significantly differ between the 15 and 50 µg HTT ASO treatments at any time point (Table 1). The mean percent changes of plasma NfL from baseline at 3 months were increases by 91.27%, 54.25% and 37.34% with PBS, 15 µg and 50 µg HTT ASO, respectively (Fig. 4e).

**Table 1:** Longitudinal plasma NfL contrasts following short-duration mHTT lowering in the brains of YAC128 mice initiated at 2 months of age

Contrast	Treatment interval (months)	MD, 95% CI (log pg/ml)	*P* value
15 µg HTT ASO-PBS	0.5	0.11, − 0.16 to 0.38	0.430
	1	0.03, − 0.21 to 0.26	0.830
	1.5	− 0.01, − 0.21 to 0.20	0.959
	2	0, − 0.25 to 0.26	0.982
	3	0.05, − 0.22 to 0.33	0.696
50 µg HTT ASO-PBS	0.5	0.15, − 0.16 to 0.47	0.338
	1	0.24, − 0.04 to 0.52	0.098
	1.5	0.21, − 0.04 to 0.46	0.093
	2	0.14, − 0.15 to 0.44	0.341
	3	0.16, − 0.2 to 0.52	0.377
50 µg HTT ASO-15 µg HTT ASO	0.5	0.04, − 0.29 to 0.38	0.796
	1	0.21, − 0.09 to 0.52	0.166
	1.5	0.22, − 0.05 to 0.48	0.106
	2	0.14, − 0.18 to 0.45	0.384
	3	0.11, − 0.27 to 0.49	0.578

MD and 95% CI of plasma NfL concentrations between PBS and HTT ASO treatment groups over a 3-month treatment interval

Cross-sectional CSF NfL concentrations were significantly increased in all treatment groups at 3 months post-treatment compared to baseline at 2 months of age. Notably, CSF NfL was significantly decreased with 50 µg HTT ASO treatment compared to PBS (Fig. 4f).

A significant positive correlation was measured between NfL concentrations in plasma and CSF at 3 months post-treatment (Fig. 4g). The relative mHTT levels in the brain following treatment with PBS, 15 µg or 50 µg HTT ASO showed a weak negative correlation with plasma NfL and a significant positive correlation with CSF NfL at this time point (Fig. 4h, i).

### Plasma and CSF NfL concentrations are stabilized in response to mHTT lowering in the brain initiated after striatal neuron loss in YAC128 mice

**Fig. 5:**
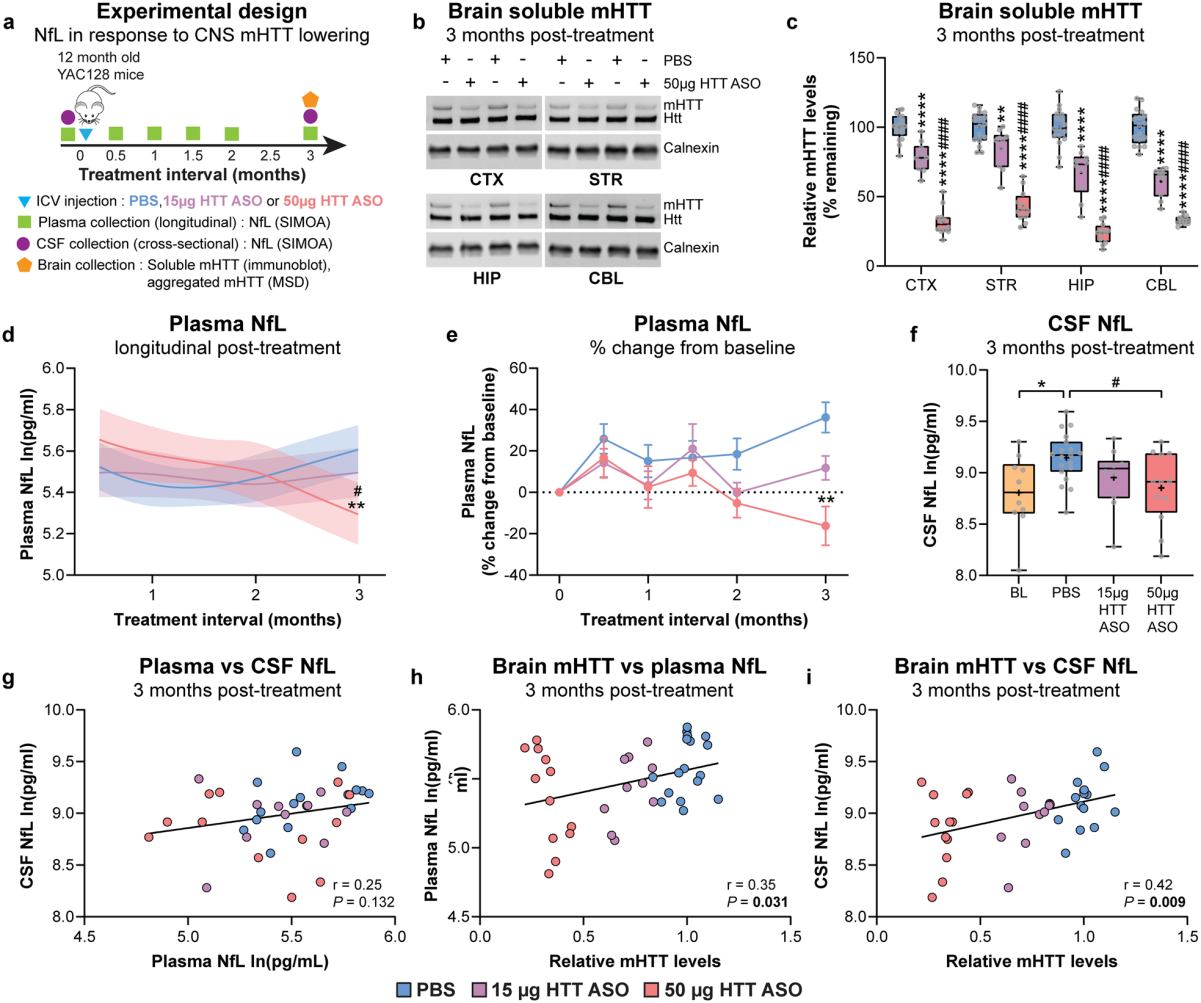
Plasma and CSF NfL concentrations are stabilized in response to mHTT lowering in the brain initiated after striatal neuron loss in YAC128 mice. **a** Schematic diagram of experimental design. **b**, **c** Representative immunoblots and quantification of soluble mHTT levels in the CTX, STR, HIP and CBL of YAC128 mice treated at 12 months of age with either PBS, 15 µg HTT ASO or 50 µg HTT ASO, and assessed 3 months post-treatment (PBS: *n* = 17; 15 µg HTT ASO: *n* = 10; 50 µg HTT ASO: *n* = 12; two-way ANOVA: treatment *P* < 0.0001, brain region *P* < 0.0001, interaction *P* = 0.0009; **P = 0.002, ****P < 0.0001 vs PBS; ^####^*P* < 0.0001 vs 15 µg HTT ASO). Levels of mHTT are normalized to PBS values. Boxes show the IQR, whiskers extend from minimum to maximum values, horizontal lines show the median, and crosses show the mean. **d** Longitudinal plasma NfL concentrations following treatment of YAC128 mice with PBS, 15 µg HTT ASO or 50 µg HTT ASO (PBS: *n* = 20; 15 µg HTT ASO: *n* = 20; 50 µg HTT ASO: *n* = 12; linear mixed-effects model: treatment *P* = 0.790, treatment × time interaction *P* = 0.001; **P = 0.002 vs PBS; ^#^*P* = 0.042 vs 15 µg HTT ASO). Models were adjusted for time from treatment and baseline NfL concentrations for each treatment condition, and fitted with a cubic spline. Solid lines denote the mean and shaded bands denote the 95% CI for each treatment condition. **e** Percent change in longitudinal plasma NfL concentrations from baseline for each animal at multiple intervals following treatment with PBS, or 15 µg or 50 µg HTT ASO (Mixed-effects model: treatment *P* = 0.057, time *P* = 0.039, treatment × time *P* = 0.015. **P = 0.003). Symbols show the mean and error bars represent SEM. **f** Cross-sectional CSF NfL concentrations in YAC128 mice at 12 months of age (BL) and 3 months post-treatment with either PBS, 15 µg HTT ASO or 50 µg HTT ASO (BL: *n* = 10; PBS: *n* = 19; 15 µg HTT ASO: *n* = 10; 50 µg HTT ASO: *n* = 12. One-way ANOVA: *P* = 0.014; *P = 0.027; ^#^*P* = 0.046; linear regression: Y = -0.0057X + 9.117, *R*^2^ = 0.16, *P* = 0.011).** g** Correlation between NfL concentrations from paired plasma and CSF samples (Pearson’s correlation:* r* = 0.25, *P* = 0.132). **h**,** i** Correlations between whole-brain mHTT level (mean mHTT from all brain regions for each animal) and (**h**) plasma (Pearson’s correlation: *r* = 0.35, *P* = 0.031) or (**i**) CSF NfL (Pearson’s correlation: *r* = 0.42, *P* = 0.009) from the same animal at 3 months post-treatment with PBS, 15 µg HTT ASO or 50 µg HTT ASO. Plasma and CSF NfL concentrations are natural log transformed

We next evaluated longitudinal plasma NfL and cross-sectional CSF NfL over a 3-month interval in response to low- and high-dose HTT ASO treatment in the brains of YAC128 mice initiated at 12 months of age (Fig. 5a). Brains were collected at 3 months post-treatment to measure the levels of full-length, soluble mHTT protein in multiple brain regions as well as aggregated mHTT load in the cortex.

We detected a dose-dependent reduction of full-length, soluble mHTT in the cortex, striatum, hippocampus and cerebellum of YAC128 mice at 3 months following HTT ASO treatment (Fig. 5b, c).

Compared to the 3-month treatment initiated at 2 months of age (Fig. 4c), both 15 µg and 50 µg HTT ASO initiated at 12 months of age resulted in a significantly greater magnitude of mHTT lowering in the brain at 3 months post-treatment (Fig. S5).

YAC128 mice develop distinct mHTT inclusion bodies in the brain from 12 months of age [75, 79], a hallmark neuropathological feature of HD [89]. We observed an age-dependent increase of mHTT inclusions (Fig. S6a) and a corresponding reduction of full-length, soluble mHTT in the cortex of YAC128 mice from 8 to 18 months of age (Fig. S6b).

A significant dose-dependent reduction of mHTT inclusion load was detected in the cortex of HTT ASO-treated YAC128 mice at 3 months post-treatment (Fig. S6c) which was accompanied by a reduction of EM48+  neuronal mHTT inclusions in the striatum with 50 µg HTT ASO (Fig. S6d). A strong positive correlation was observed between the relative soluble mHTT and the aggregated mHTT levels in the cortex following short-duration mHTT lowering initiated at 12 months of age (Fig. S6e).

A significant treatment-by-time interaction on longitudinal plasma NfL concentrations were observed over a 3-month interval in response to HTT ASO treatment initiated at 12 months of age (Fig. S7, model including baseline NfL concentrations; Fig. 5d, model adjusted for baseline NfL concentrations). Plasma NfL concentrations were not significantly changed at any time point in response to treatment with 15 µg HTT ASO compared to PBS over the treatment interval (Table 2). In contrast, a trend towards increased plasma NfL was measured in response to 50 µg HTT ASO treatment up to 2 months post-treatment, followed by a significant reduction at the 3-month time point compared to PBS and 15 µg HTT ASO treatment. Plasma NfL concentrations were increased by 36.20% with PBS and 11.81% with 15 µg HTT ASO, and decreased by 16.18% with 50 µg HTT ASO from baseline, at 3 months post-treatment (Fig. 5e).

**Table 2:** Longitudinal plasma NfL contrasts following short-duration mHTT lowering in the brains of YAC128 mice initiated at 12 months of age

Contrast	Treatment interval (months)	MD, 95% CI (log pg/ml)	*P* value
15 µg HTT ASO-PBS	0.5	− 0.03, − 0.2 to 0.14	0.718
	1	0.05, − 0.10 to 0.20	0.533
	1.5	0.04, − 0.10 to 0.17	0.597
	2	− 0.03, − 0.19 to 0.14	0.755
	3	− 0.11, − 0.28 to 0.06	0.185
50 µg HTT ASO-PBS	0.5	0.13, − 0.06 to 0.32	0.182
	1	0.14, − 0.03 to 0.32	0.101
	1.5	0.12, − 0.03 to 0.27	0.122
	2	0.04, − 0.15 to 0.22	0.700
	3	− 0.31, − 0.51 to − 0.12	**0.002**
50 µg HTT ASO-15 µg HTT ASO	0.5	0.16, − 0.03 to 0.35	0.096
	1	0.10, − 0.07 to 0.27	0.264
	1.5	0.08, − 0.07 to 0.24	0.267
	2	0.06, − 0.12 to 0.24	0.506
	3	− 0.20, − 0.39 to − 0.01	**0.042**

MD and 95% CI of plasma NfL concentrations between PBS and HTT ASO treatment groups over a 3-month treatment interval. *P *values that indicate significant changes are bolded

Cross-sectional CSF NfL concentrations were significantly increased with PBS but not with 15 µg or 50 µg HTT ASO at 3 months post-treatment compared to the baseline level at 12 months of age (Fig. 5f). In addition, CSF NfL concentrations were significantly decreased with 50 µg HTT ASO compared to PBS at 3 months post-treatment.

A weak association was observed between plasma and CSF NfL concentrations at 3 months post-treatment (Fig. 5g). The relative mHTT levels in the brain showed a significant positive correlation with both plasma NfL and CSF NfL following treatment with PBS, 15 µg or 50 µg HTT ASO at this time point (Fig. 5h, i).

### Sustained reduction of mHTT in the brains of YAC128 mice initiated prior to overt striatal neuron loss stabilizes increase of plasma and CSF NfL

**Fig. 6:**
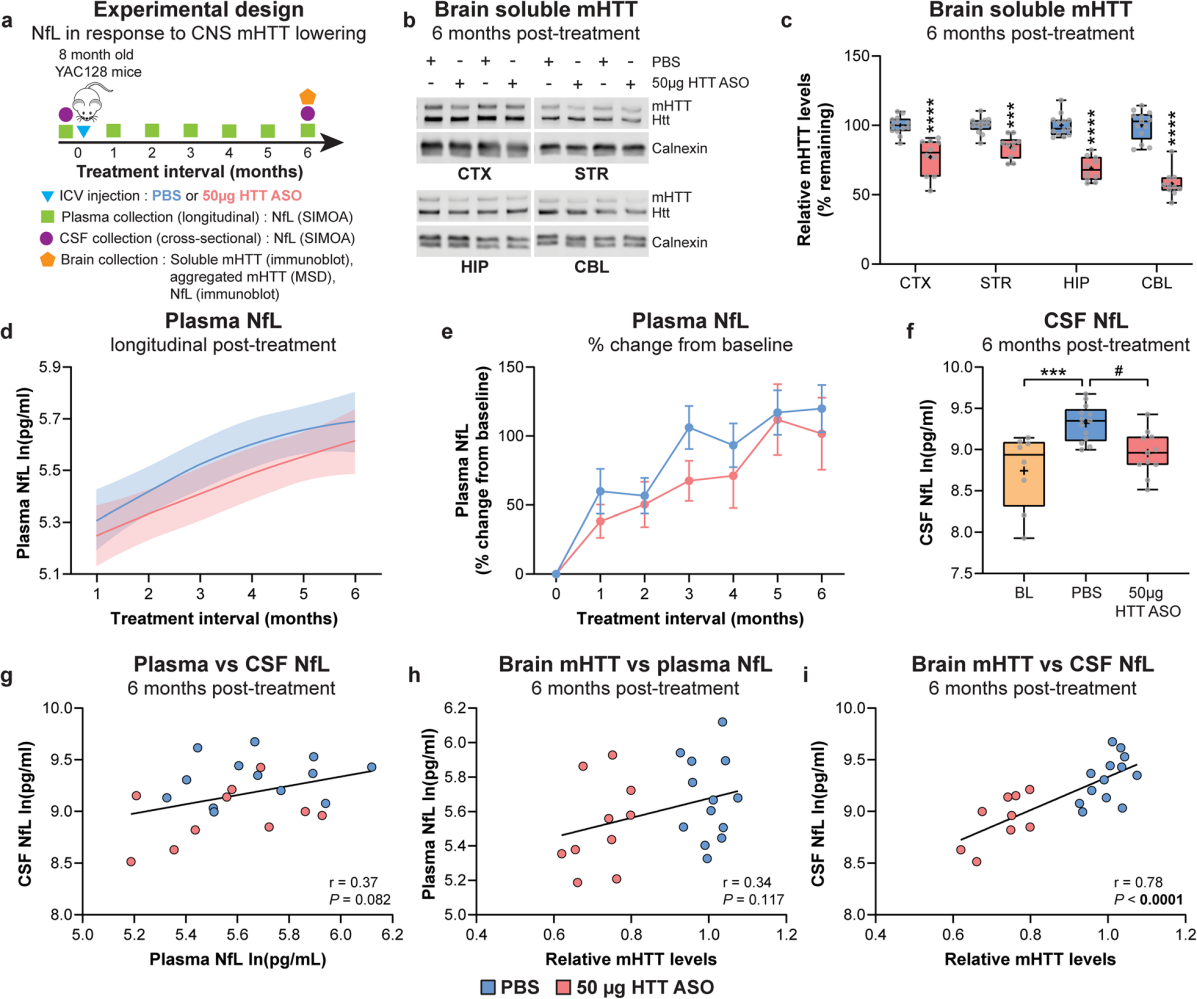
Sustained reduction of mHTT in the brains of YAC128 mice initiated prior to overt striatal neuron loss stabilizes increases of plasma and CSF NfL. **a** Schematic diagram of experimental design. **b**, **c** Representative immunoblots and quantification of relative soluble mHTT levels in the CTX, STR, HIP and CBL of YAC128 mice treated at 8 months of age with either PBS or 50 µg HTT ASO, and assessed 6 months post-treatment (PBS: *n* = 13; 50 µg HTT ASO: *n* = 10; two-way ANOVA: treatment *P* < 0.0001, brain region *P* < 0.0001, interaction *P* < 0.0001; ***P = 0.0003, ****P < 0.0001). Levels of mHTT are normalized to PBS values for each brain region. Boxes show the IQR, whiskers extend from minimum to maximum values, horizontal lines show the median, and crosses show the mean. **d** Longitudinal plasma NfL concentrations following treatment with either PBS or 50 µg HTT ASO (PBS: *n* = 13; 50 µg HTT ASO: *n* = 13; linear mixed-effects model: treatment *P* = 0.056, treatment × time interaction *P* = 0.934). Models were adjusted for time from treatment and baseline NfL concentrations for each treatment condition, and fitted with a cubic spline. Solid lines denote the mean and shaded bands denote 95% CI for each treatment condition. **e** Percent change in longitudinal plasma NfL concentrations from baseline at different intervals following treatment with either PBS or 50 µg HTT ASO (Mixed-effects model: treatment *P* = 0.411, time *P* < 0.0001, treatment × time *P* = 0.565). Symbols show the mean and error bars represent SEM. **f** Cross-sectional CSF NfL concentrations at 8 months of age (BL) and 6 months post-treatment with either PBS or 50 µg HTT ASO (BL: *n* = 8; PBS: *n* = 13; 50 µg HTT ASO: *n* = 11. One-way ANOVA: *P* = 0.0007; ***P = 0.0007; ^#^*P* = 0.020). **g** Correlation between NfL concentrations from paired plasma and CSF samples (Pearson’s correlation: *r* = 0.37, *P* = 0.082). **h**, **i** Correlations between the whole-brain mHTT level (mean mHTT from all brain regions for each animal) and (**h**) plasma (Pearson’s correlation:* r* = 0.34, *P* = 0.117) or (**i**) CSF NfL (Pearson’s correlation: *r* = 0.78, *P* < 0.0001) from the same animal at 6 months post-treatment with PBS or 50 µg HTT ASO. Plasma and CSF NfL concentrations are natural log transformed

We next evaluated longitudinal plasma NfL and cross-sectional CSF NfL over a 6-month interval in response to high-dose HTT ASO treatment in the brains of YAC128 mice initiated at 8 months of age (Fig. 6a). Brains were collected at 6 months post-treatment to assess target engagement of full-length, soluble mHTT in multiple brain regions, aggregated mHTT in the cortex, and NfL protein levels in the striatum.

The levels of full-length, soluble mHTT were significantly reduced in the cortex, striatum, hippocampus and cerebellum of YAC128 mice with 50 µg HTT ASO compared to PBS at the 6-month treatment interval (Fig. 6b, c). Compared to long-duration mHTT lowering with 50 µg HTT ASO initiated at 2 months of age, the relative magnitude of mHTT lowering in the brain with this dose was significantly greater when initiated at 8 months of age (Fig. S8).

We also detected a significant reduction of aggregated mHTT in the cortex following treatment with 50 µg HTT ASO (Fig. S9a), and a strong positive correlation between the relative soluble mHTT (Fig. 6c) and the aggregated mHTT levels at 6-month post-treatment (Fig. S9b).

A strong trend towards a significant treatment effect on plasma NfL concentrations was observed over the 6-month interval in response to 50 µg HTT ASO treatment initiated at 8 months of age (Fig. S10, model including baseline NfL concentration; Fig. 6d, model adjusted for baseline NfL concentration). Plasma NfL concentrations were decreased with 50 µg HTT ASO at all time points over the 6-month treatment interval compared to PBS, showing the greatest reduction at 3 and 4 months post-treatment (Table 3). The mean percent change of plasma NfL at 3 months post-treatment from baseline was increased by 106.25% with PBS, compared to 67.50% with 50 µg HTT ASO (*P* = 0.408; Fig. 6e). At 6 months post-treatment, the increase from baseline was 119.99% with PBS compared to 101.72% with 50 µg HTT ASO.

**Table 3:** Longitudinal plasma NfL contrasts following long-duration mHTT lowering in the brains of YAC128 mice initiated at 8 months of age

Contrast	Treatment interval (months)	MD, 95% CI (log pg/ml)	*P* value
50 µg HTT ASO-PBS	1	− 0.06, − 0.22 to 0.11	0.497
	2	− 0.09, − 0.22 to 0.04	0.182
	3	− 0.11, − 0.25 to 0.03	0.110
	4	− 0.11, − 0.25 to 0.02	0.104
	5	− 0.10, − 0.23 to 0.04	0.148
	6	− 0.07, − 0.24 to 0.10	0.403

MD and 95% CI of plasma NfL concentrations between PBS and HTT ASO treatment groups over a 6-month treatment interval

Cross-sectional CSF NfL concentrations were significantly increased with PBS but not with 50 µg HTT ASO at 6 months post-treatment compared to baseline level at 8 months of age (Fig. 6f). Notably, CSF NfL concentrations were significantly decreased with 50 µg HTT ASO compared to PBS at the 6-month treatment interval.

A moderate positive correlation was observed between plasma and CSF NfL concentrations at 6 months post-treatment. Relative mHTT levels in the brain showed a moderate association with plasma NfL concentration and a strong positive correlation with CSF NfL following treatment with PBS or 50 µg HTT ASO at this time point (Fig. 6g–i).

NfL protein levels in the striatum were significantly reduced following treatment with 50 µg HTT ASO compared to PBS at 6 months post-treatment. Notably, striatal NfL showed a strong positive correlation with mHTT levels in the striatum, as well as moderate positive correlations with both plasma and CSF NfL levels (Fig. S11).

## Discussion

Therapies aimed at lowering mHTT in the CNS have emerged as promising approaches for treating HD. Preclinical studies have shown that lowering mHTT in the brain can prevent regional forebrain atrophy and neurodegeneration in HD rodent models [12–14]. These findings have accelerated clinical development and therapeutic trials of various HTT-lowering modalities for HD. NfL has been validated in multiple well-powered studies as a sensitive and robust biomarker for monitoring disease severity, progression and prognostic outcome in HD [27–31] and has been included as an exploratory endpoint in clinical trials for HD [21, 22, 90, 91]. However, little is known of whether NfL in biofluids could serve as a response biomarker to assess efficacy of candidate disease-modifying therapies for HD. To address this gap in knowledge, we investigated NfL concentrations in CSF and plasma in response to mHTT lowering in the brains of YAC128 mice, as well as the impacts of timing and magnitude of mHTT lowering on NfL concentrations in biofluids. We chose the YAC128 model, in part because it permits selective lowering of human mutant HTT without altering the expression of the animal’s two wild-type alleles, excluding wild-type HTT deficiency as a mechanism for possible NfL alteration.

We characterized the natural history of NfL in plasma and CSF from YAC128 and WT littermate control mice. Concentrations of NfL in CSF were orders of magnitude higher compared to those measured in plasma at any given age, as reported in HD mutation carriers [27, 29]. We observed an age-dependent increase of NfL in plasma and CSF from both genotypes, which is consistent with the reported correlation of NfL in biofluids with age in healthy individuals [92] and HD mutation carriers [29]. Moreover, longitudinal plasma NfL concentrations increased with age at a greater rate in YAC128 mice compared to WT littermate controls, which is consistent with the reported rise of NfL concentrations in biofluids with HD severity and progression [28, 29]. Notably, plasma and CSF NfL concentrations in YAC128 were significantly elevated compared to WT mice from 9 months of age, which coincides with the reported 9% loss of total striatal neurons in YAC128 mice at this age [75]. We also detected non-significant increases of CSF and plasma NfL at 2 and 6 months of age in YAC128 mice, which could reflect the modest striatal atrophy reported from 3 months of age in this model by MRI [77]. Taken together, these data provide evidence that biofluid NfL concentrations in YAC128 mice may accurately model the changes observed in people with HD.

Importantly, a strong positive correlation of NfL concentrations between paired plasma and CSF concentrations was found at 12 months of age in WT and YAC128 mice. CSF flow and exchange with the blood compartment plays an important role in the clearance of solutes from the brain interstitial space [93]. The association of NfL concentrations between biofluids suggests that both CSF and plasma NfL reflect the same pathophysiological perturbations in the brains of YAC128 mice. We observed that CSF NfL showed superior discrimination of YAC128 and WT control mice at 12 months of age, suggesting that CSF NfL may be a more robust marker of neurodegeneration and therefore more sensitive for measuring the response to mHTT lowering in the brains of YAC128 mice. However, CSF collection from the cisterna magna of mice is typically a terminal procedure, limiting our study to cross-sectional analyses of CSF NfL concentrations. In contrast, plasma collection via saphenous vein is relatively non-invasive and can be performed repeatedly from the same animal, allowing for monitoring of longitudinal NfL dynamics.

QA, a glutamate agonist, induces excitotoxicity by binding and potentiating NMDA receptors in neurons [94]. We evaluated NfL in matched CSF and plasma samples in response to QA-induced acute neurodegeneration in the striatum of YAC128 mice to assess the temporal dynamics of NfL changes in this model. CSF NfL was significantly elevated in response to QA treatment compared to PBS at both 3- and 7-day intervals. Interestingly, CSF NfL concentrations were ~ 8 times higher at the earlier time point (3 days: 21,711 pg/ml vs 7 days: 2616 pg/ml), suggesting that most neurons in the striatum exposed to QA have degenerated by day 7. This finding is supported by the significantly reduced *Nefl* expression at both time points and increased NfL protein levels in the injected striatum at the 3-day interval following QA injection. In contrast, plasma NfL concentrations were significantly increased at 7 but not 3 days after QA administration, where a strong negative correlation was measured between CSF and plasma NfL. Together these data suggest that NfL is rapidly released from degenerating neurons in the brain to CSF in response to neurotoxic insult, and more slowly cleared from CSF to the blood compartment. The delayed increase in plasma NfL following neuronal injury is also consistent with the reported elevated serum NfL level in boxers 7–10 days after a bout [59]. The sensitive, robust and relatively rapid response of NfL in biofluids to acute neurotoxicity in the brain suggests that NfL could serve as a safety biomarker in early-phase clinical trials to flag candidate drugs that are poorly tolerated and adversely affect neuronal health, as well as to help guide appropriate dosing and treatment paradigms. As an example, the transient dose-dependent increase of CSF NfL in response to tominersen treatment in the phase I/II trial [19], which was subsequently observed with the dosing regimen of 120 mg tominersen every 8 weeks in the GENERATION HD1 phase III trial [20], could have been used as an early indicator for potential neurotoxic properties of the drug at higher doses and/or more frequent treatment intervals.

Intrathecal delivery of ASOs into the subarachnoid space of the spinal cord has been used in clinical trials for HD [15–20] and other neurodegenerative diseases, such as SMA [95]. This route of administration bypasses the blood–brain barrier, but drugs are subject to rapid efflux from the CSF and limited diffusion into the brain parenchyma. ASOs delivered into the CSF result in target RNA suppression in many cell types of the brain, including neurons and glia [96]. Preclinical studies in rhesus macaques have demonstrated that intrathecally-delivered ASOs distribute unevenly throughout the brain, resulting in a concentration gradient where ASO levels are highest in brain regions proximal to CSF, and lower in areas more distal from the CSF compartment [12, 13]. Post-mortem analysis of the brain from an HD patient treated with tominersen revealed a similar distribution gradient of ASO (Dr. Blair Leavitt; CHDI HDTC 2023 presentation).

ICV delivery into the CSF of the lateral ventricle can be reliably performed in mice and was used in our study as an analogous route to the intrathecal administration employed in clinical trials to deliver ASOs to the CNS. We and others have previously shown that ICV injection of *HTT*-targeted ASOs in rodent models of HD are distributed broadly and lead to global mHTT lowering throughout the brain [13, 82, 97]. Consistent with these reports, we found that a single unilateral ICV injection with either 15 or 50 µg HTT ASO, initiated at 2 or 12 months of age in YAC128 mice, was well tolerated and resulted in dose-dependent reduction of full-length, soluble mHTT in multiple brain structures (e.g., cortex, striatum, hippocampus and cerebellum) at a 3-month post-treatment interval. Moreover, high-dose HTT ASO treatment initiated at 8 months of age resulted in a sustained reduction of soluble mHTT throughout the brain up to 6 months post-treatment. Notably, we observed that the magnitude of mHTT reduction throughout the brain was greater when treatment was initiated in older YAC128 mice, when HD-like phenotypes in this model are more severe. Dysfunction of the glymphatic system has been reported in HD [98, 99], which we hypothesize may contribute to reduced clearance of ASOs and other solutes (e.g., mHTT) from the brain parenchyma at older ages in YAC128 mice. This is further supported by reports showing that the glymphatic function is impaired in neurodegenerative diseases (reviewed in [100]) and in the aging brain [101, 102]. Our findings suggest that disease severity and age may influence the distribution and/or the uptake of ASO in the brain, and may have implications for the design of dosing paradigms for HD clinical trials.

Inclusion bodies composed of aggregated mHTT within neurons of the CNS are a hallmark neuropathological feature of HD [89]. Widespread mHTT inclusion bodies have been reported in the cortex and striatum of YAC128 mice from as early as 6 months of age [103], where the number and the size of mHTT aggregates increase with age [75, 79]. Consistent with these findings, we detected an age-dependent increase in mHTT inclusion load in the cortex of YAC128 mice from 8 to 18 months of age, which was coincident with a reduction of full-length, soluble mHTT. These data highlight an age-dependent transition from soluble to aggregated mHTT in this mouse model.

As part of our study, we also investigated the impact of HTT ASO treatment in the CNS on mHTT inclusion load in the brains of YAC128 mice. We found that mHTT lowering initiated early (at 8 months) and late (at 12 months) during mHTT inclusion formation led to a significant reduction in mHTT inclusion load in the cortex, which correlated strongly with the level of soluble mHTT in paired cortical samples. Other groups have previously reported sustained reduction of mHTT aggregate load in YAC128 mice following treatment with virally-encoded RNAi modalities targeting *HTT* [103–105]. However, to our knowledge, this is the first report to demonstrate that transient mHTT lowering in CNS using an ASO leads to a reduction of mHTT aggregates in the brain.

Based on the extensive phenotypic characterization of the YAC128 model [75–81], we designed our study to evaluate the levels of NfL in biofluids in response to mHTT lowering in the brain initiated at different ages to model therapeutic intervention during the premanifest (pre-symptomatic) and manifest (symptomatic) stages of HD.

YAC128 mice at 2 months of age model a pre-symptomatic stage of disease and do not exhibit overt behavioural phenotypes, regional brain atrophy, or neurodegeneration. Consistent with this finding, we did not detect a significant genotypic difference in plasma or CSF NfL concentrations at this age. HTT ASO treatment initiated at 2 months of age resulted in a dose-dependent reduction of soluble mHTT throughout the brain (~ 18% and 54% with 15 and 50 µg HTT ASO, respectively) without any measurable induction of gliosis. Plasma NfL concentrations were not significantly changed in response to mHTT lowering over the 3-month treatment interval. In contrast, a dose-dependent reduction of CSF NfL was measured following HTT ASO treatment, where there was a significant positive correlation between the magnitude of mHTT lowering in the brain and corresponding CSF NfL concentrations. Our data suggest that CSF NfL is a more sensitive response biomarker when intervention is initiated prior to overt brain atrophy and neurodegeneration in YAC128 mice. This may be due to the higher baseline levels of NfL in CSF compared to plasma at the pre-symptomatic stage of disease, thus providing a greater window to detect a decrease of NfL.

YAC128 mice at 12 months of age model an advanced symptomatic stage of HD, characterized by behavioural abnormalities, robust regional forebrain atrophy, and striatal neuron loss [75–78, 81]. At this age, CSF and plasma NfL concentrations were significantly elevated compared to WT littermate control mice. Treatment of 12-month-old YAC128 mice with 15 or 50 µg HTT ASO resulted in a sustained, dose-dependent reduction of soluble (27% and 67%, respectively) and aggregated mHTT (17% and 28%, respectively) in the brain at 3 months post-treatment. Notably, we observed an interaction between treatment and time on plasma NfL concentrations, where high-dose HTT ASO treatment resulted in a significant reduction of absolute plasma NfL concentrations and mean percent change from baseline compared to PBS treatment by 3 months post-treatment. Moreover, CSF NfL concentrations were significantly reduced following treatment with 50 µg HTT ASO compared to PBS at 3 months post-treatment, and were not significantly increased from baseline concentrations. Importantly, there was a significant positive correlation between the relative mHTT levels in the brain and the NfL concentrations in both plasma and CSF, demonstrating a relationship between the magnitude of mHTT lowering in the brain and the response of NfL in biofluids. Our data suggest that both plasma and CSF NfL are robust response biomarkers when mHTT lowering in the brain is initiated after marked loss of striatal neuron populations in YAC128 mice. We postulate that the stabilization of NfL concentrations in both biofluids may reflect slowing of neurodegeneration and cell loss at this advanced stage of disease.

YAC128 mice at 8 months of age model an early/mid symptomatic stage of HD, where animals display clear behavioural and neuropathological phenotypes, although striatal cell loss may be absent [75, 77, 78, 81]. We have previously demonstrated that the ASO-mediated mHTT lowering initiated at 6 months in a humanized mouse model of HD ameliorates forebrain atrophy and prevents striatal neurodegeneration at 12 months of age [13]. Concentrations of CSF and plasma NfL showed a trend toward an increase in YAC128 compared to WT littermate control mice at 8 months of age. High-dose HTT ASO treatment of YAC128 mice initiated at this age resulted in a potent and sustained reduction of soluble (~ 28%) and aggregated mHTT (~ 23%) in the brain at the 6-month interval. This magnitude of mHTT lowering was accompanied by a stabilization of plasma NfL increase compared to PBS treatment over this treatment interval, where the strongest effect was observed at 3–4 months post-treatment. Moreover, CSF NfL concentrations in animals treated with 50 µg HTT ASO were significantly reduced at 6 months post-treatment compared to those treated with PBS, but were not significantly increased from concentrations at baseline. Our data demonstrate a measurable stabilization of NfL increases in both plasma and CSF in response to mHTT lowering in the brains of YAC128 mice initiated at an age modeling an early-to-mid symptomatic stage of HD. We postulate that this stabilization of NfL may reflect improved neuronal health over a period when active neurodegeneration is occurring in this model.

A limitation of this study is that we did not validate that the measured changes of NfL in response to mHTT lowering in the CNS are directly related to improvements of HD-like behavioural and neuropathological phenotypes. Additional studies incorporating neuropathological analyses and/or imaging-based measures are needed to investigate the relationship between structural changes in the brain and NfL concentrations in biofluids in response to mHTT lowering in the CNS. These studies can help further inform whether NfL could be useful as a response biomarker for monitoring therapeutic efficacy related to neuronal preservation in HD.

Another potential limitation of our study was the use of a single ICV injection to deliver HTT ASO, rather than employing repeated or continuous infusion methods. Lowering of mHTT in the CNS with ASOs is transient, where the duration of reduction following a single dose typically lasts on the order of months [13, 106]. An efficacious treatment aimed at modifying the course of HD will therefore likely necessitate repeated administration of the drug to sustain mHTT lowering over extended periods of time. We observed that treatment with both 15 and 50 µg HTT ASO resulted in a peak mHTT reduction at ~ 1 month in all brain regions, with levels of mHTT gradually returning to baseline in a dose-dependent manner over a 6-month time frame. Additional preclinical studies are needed in which HTT ASO is administered repeatedly to more accurately model treatment paradigms in clinical trials for HD. This will help us better understand the dynamics of NfL in biofluids in response to sustained mHTT lowering in the CNS.

## Conclusions

In this study, we present evidence that NfL in biofluids responds to mHTT lowering in the CNS, with both the magnitude of mHTT reduction and the timing of intervention during the disease course influencing this response in the CSF and plasma. Our findings support further investigations of NfL as a response biomarker for evaluating the efficacy of disease-modifying therapies for HD.

## Supplementary Information

**Additional file 1**: **Fig. S1**. Natural history of plasma and CSF NfL in YAC128 mice **Fig. S2**. Extended time-course of mHTT lowering in the CNS of YAC128 mice. **Fig. S3**. Treatment with HTT ASO does not induce increased Gfap expression in the brain of YAC128 mice. **Fig. S4**. Plasma NfL concentrations following short duration mHTT lowering in the brain of YAC128 mice initiated at 2 months of age. **Fig. S5**. Short duration treatment with HTT ASO initiated at older ages in YAC128 mice leads to a higher magnitude of mHTT lowering in the brain. **Fig. S6**. Short duration mHTT lowering initiated at 12 months of age reduces mHTT inclusion load in the brain of YAC128 mice. **Fig. S7**. Plasma NfL concentrations following short duration mHTT lowering in the brain of YAC128 mice initiated at 12 months of age. **Fig. S8**. Long duration treatment with HTT ASO initiated at older ages in YAC128 mice leads to a higher magnitude of mHTT lowering in the brain. **Fig. S9**. Long duration mHTT lowering initiated at 8 months of age reduces mHTT inclusion load in the brain of YAC128 mice. **Fig. S10**. Plasma NfL concentrations following long duration mHTT lowering in the brain of YAC128 mice initiated at 8 months of age. **Fig. S11**. NfL protein levels are reduced in the striatum following long duration mHTT lowering in the brain of YAC128 mice.

**Additional file 2**: Immunoblot of NfL levels in the striatum of YAC128 mice at days 3 and 7 following intrastriatal injection with PBS or QA.

## Data Availability

The datasets used and/or analysed during the current study are available from the corresponding author on reasonable request.

## Abbreviations

ALS: Amyotrophic lateral sclerosis
ASO: Antisense oligonucleotide
AUC: Area under the curve
CNS: Central nervous system
CSF: Cerebrospinal fluid
HD: Huntington disease
i.p.: Intraperitoneal
mHTT: Mutant huntingtin
MS: Multiple sclerosis
MSD: Mesoscale discovery
NfL: Neurofilament light chain
PFA: Paraformaldehyde
QA: Quinolinic acid
ROC: Receiver operating characteristic
qPCR: Quantitative polymerase chain reaction
SIMOA: Single molecule array
SMA: Spinal muscular atrophy
