# Supplementary material for: Elevated plasma and CSF neurofilament light chain concentrations are stabilized in response to mutant huntingtin lowering in the brains of Huntington’s disease mice
Source: Transl Neurodegener. 2024 Oct 8;13:50. doi: 10.1186/s40035-024-00443-8 (PMC11460072; doi:10.1186/s40035-024-00443-8)
Supplement: Supplementary file 1 — Additional file 1: Fig. S1. Natural history of plasma and CSF NfL in YAC128 mice Fig. S2. Extended time-course of mHTT lowering in the CNS of YAC128 mice. Fig. S3. Treatment with HTT ASO does not induce increased Gfap expression in the brain of YAC128 mice. Fig. S4. Plasma NfL concentrations following short duration mHTT lowering in the brain of YAC128 mice initiated at 2 months of age. Fig. S5. Short duration treatment with HTT ASO initiated at older ages in YAC128 mice leads to a higher magnitude of mHTT lowering in the brain. Fig. S6. Short duration mHTT lowering initiated at 12 months of age reduces mHTT inclusion load in the brain of YAC128 mice. Fig. S7. Plasma NfL concentrations following short duration mHTT lowering in the brain of YAC128 mice initiated at 12 months of age. Fig. S8. Long duration treatment with HTT ASO initiated at older ages in YAC128 mice leads to a higher magnitude of mHTT lowering in the brain. Fig. S9. Long duration mHTT lowering initiated at 8 months of age reduces mHTT inclusion load in the brain of YAC128 mice. Fig. S10. Plasma NfL concentrations following long duration mHTT lowering in the brain of YAC128 mice initiated at 8 months of age. Fig. S11. NfL protein levels are reduced in the striatum following long duration mHTT lowering in the brain of YAC128 mice. [file 40035_2024_443_MOESM1_ESM.docx]

**Additional file 1**

**
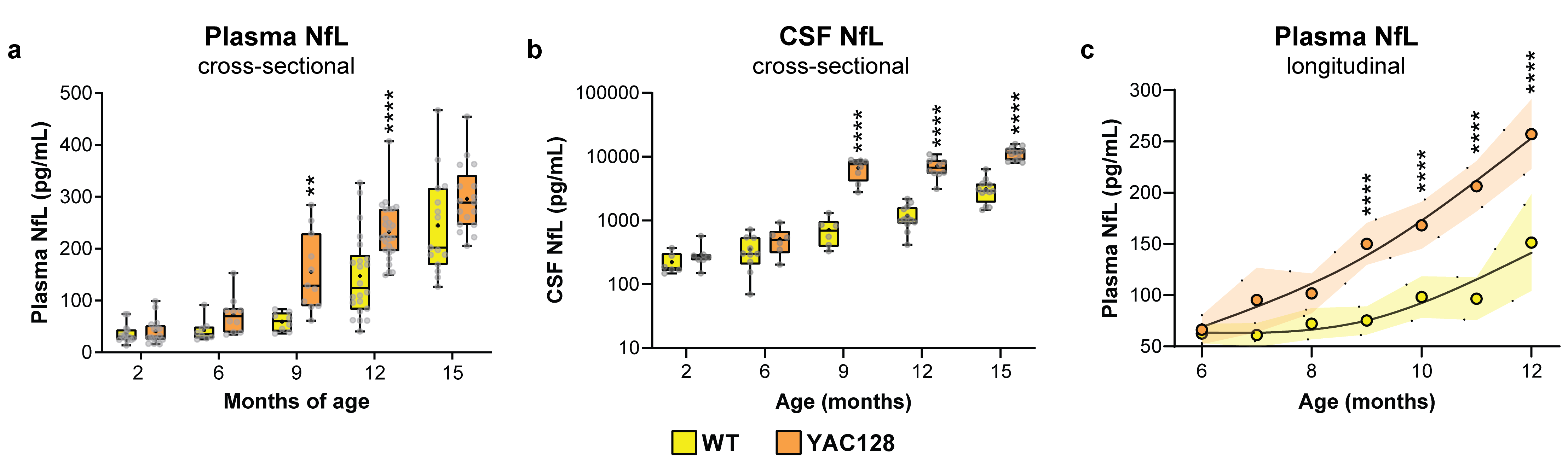
**

**Fig. S1.** **Natural history of plasma and CSF NfL concentrations in YAC128 mice. a, b** Cross-sectional NfL concentrations (pg/ml) in (**a**) plasma (2 months: *n =* 14 WT, *n =* 16 YAC128; 6 months: *n =* 8 WT, *n =* 10 YAC128; 9 months: *n =* 8 WT, *n =* 11 YAC128; 12 months: *n =* 22 WT, *n =* 26 YAC128; 15 months: *n =* 15 WT, *n =* 17 YAC128. Two-way ANOVA: genotype *P* < 0.0001, age *P* < 0.0001, interaction *P* = 0.029. ***P =* 0.004, *****P* < 0.0001) and (**b**) CSF (2 months: *n =* 6 WT, *n =* 7 YAC128; 6 months: *n =* 10 WT, *n =* 6 YAC128; 9 months: *n =* 6 WT, *n =* 8 YAC128; 12 months: *n =* 12 WT, *n =* 10 YAC128; 15 months: *n =* 12 WT, *n =* 12 YAC128. Two-way ANOVA: genotype *P* < 0.0001, age *P* < 0.0001, interaction *P* < 0.0001. *****P* < 0.0001) from YAC128 and WT littermate control mice. CSF NfL concentrations are shown on the log scale. Boxes show the IQR, whiskers extend from minimum to maximum values, horizontal lines show the median, and crosses show the mean. **c** Longitudinal plasma NfL concentrations from 6 to 12 months of age in WT and YAC128 mice (WT: *n =* 21, YAC128: *n =* 19. Mixed-effect analysis: genotype *P* < 0.0001, age *P* < 0.0001, genotype x age *P* < 0.0001. *****P* < 0.0001). A spline was fit to mean values for each genotype where shaded bands denote 95% CI. Plasma and CSF NfL concentrations are untransformed.

CI: confidence interval, IQR: interquartile range, WT: wild type


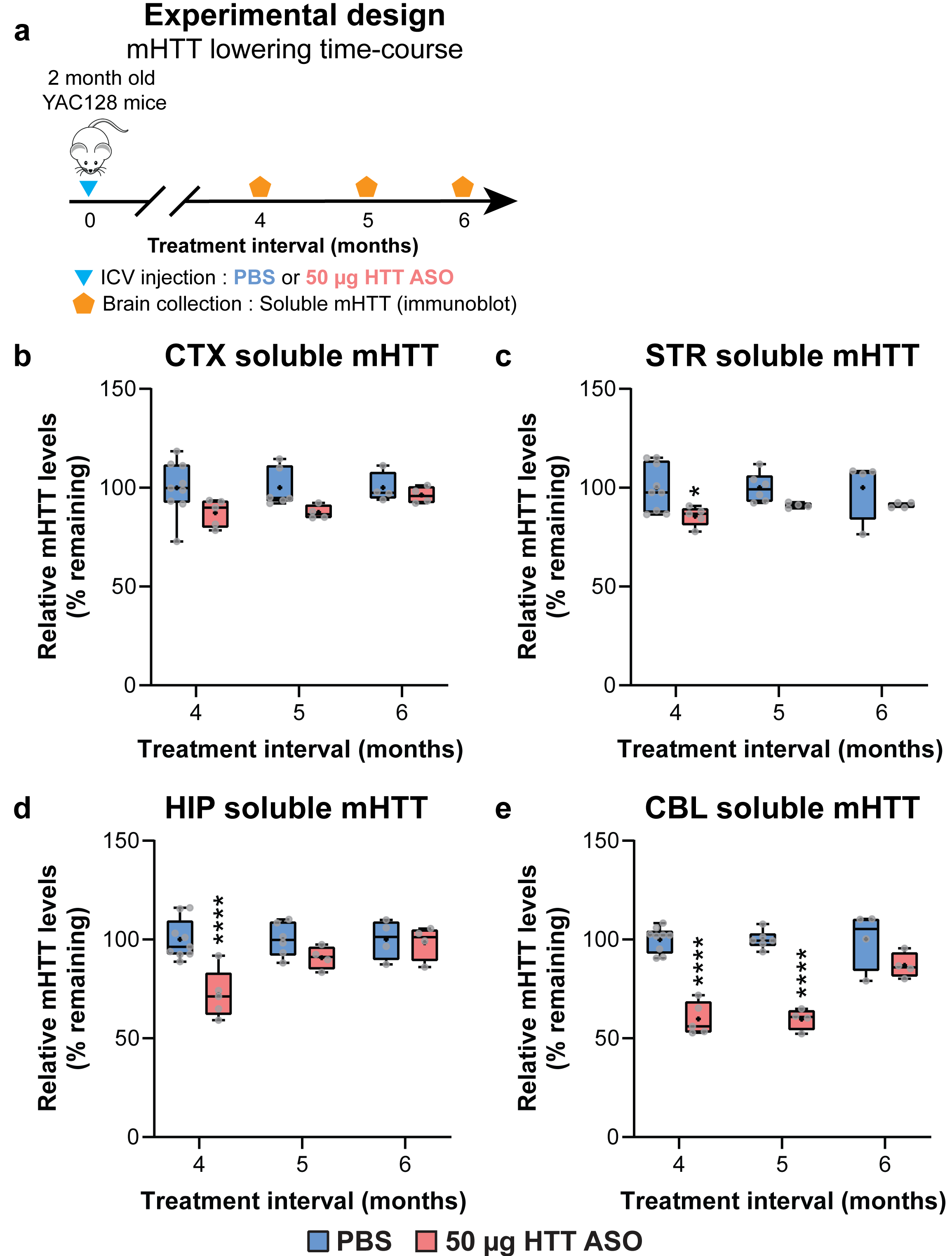


**Fig. S2.** **Extended time-course of mHTT lowering in the brains of YAC128 mice.** **a** Schematic diagram of experimental design (4 months: *n =* 9 PBS, *n =* 5 50 µg HTT ASO; 5 months: *n =* 6 PBS, *n =* 4 50 µg HTT ASO; 6 months: *n =* 4 PBS, *n =* 4 50 µg HTT ASO). **b-e** Quantification of relative soluble mHTT protein levels in the (**b**) cortex (Two-way ANOVA: treatment *P* = 0.012, interval *P* = 0.539, interaction *P* = 0.539), (**c**) striatum (Two-way ANOVA: treatment *P* = 0.004, interval *P* = 0.723, interaction *P* = 0.723. **P* = 0.030), (**d**) hippocampus (Two-way ANOVA: treatment *P* = 0.001, interval *P* = 0.012, interaction *P* = 0.012. *****P* < 0.0001), and (**e**) cerebellum (Two-way ANOVA treatment *P* < 0.0001, interval *P* = 0.001, interaction *P* = 0.001. *****P* < 0.0001) up to 6 months post-treatment with either PBS or 50 µg HTT ASO. Levels of mHTT in all brain regions are shown relative to PBS values at each respective time point. Boxes show the IQR, whiskers extend from minimum to maximum values, horizontal lines show the median, and crosses show the mean.

ASO: antisense oligonucleotide, CBL: cerebellum, CTX: cortex, HIP: hippocampus, ICV: intracerebroventricular, IQR: interquartile range, mHTT: mutant huntingtin, PBS: phosphate-buffered saline, STR: striatum


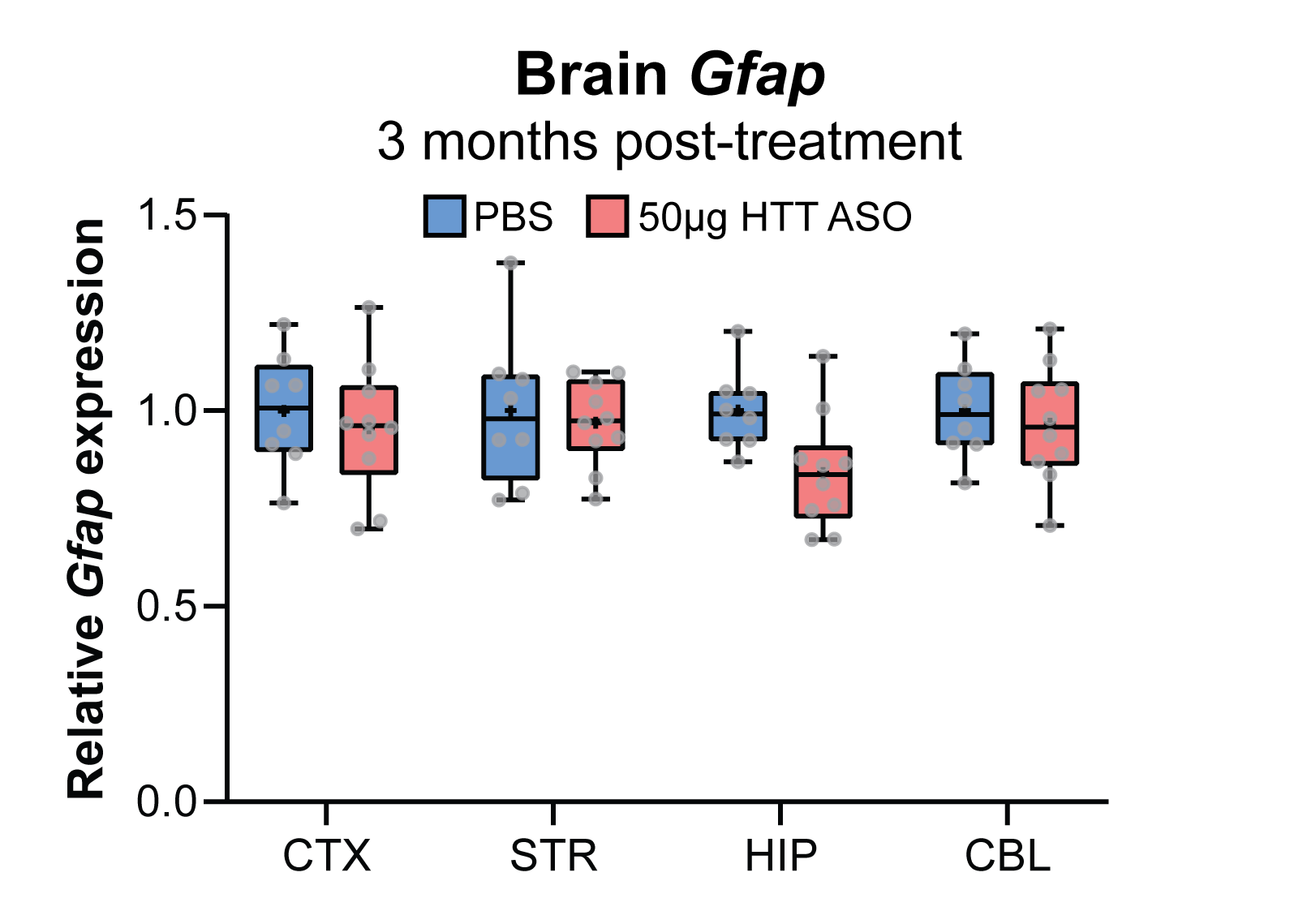


**Fig. S3.** **Treatment with HTT ASO does not induce increased *Gfap* expression in the brains of YAC128 mice.** Relative expression of *Gfap* transcript in the brain of YAC128 mice treated at 2 months of age with either PBS or 50 µg HTT ASO and assessed 1-month post-treatment, at 3 months of age (PBS: *n =* 8; 50 µg HTT ASO: *n =* 10. Two-way ANOVA: treatment *P* = 0.057, brain region *P* = 0.497, interaction *P* = 0.497). *Gfap* transcript levels in all brain regions are shown relative to PBS values for each respective brain region. Boxes show the IQR, whiskers extend from minimum to maximum values, horizontal lines show the median, and crosses show the mean.

ASO: antisense oligonucleotide, CBL: cerebellum, CTX: cortex, HIP: hippocampus, IQR: interquartile range, PBS: phosphate-buffered saline, STR: striatum

**
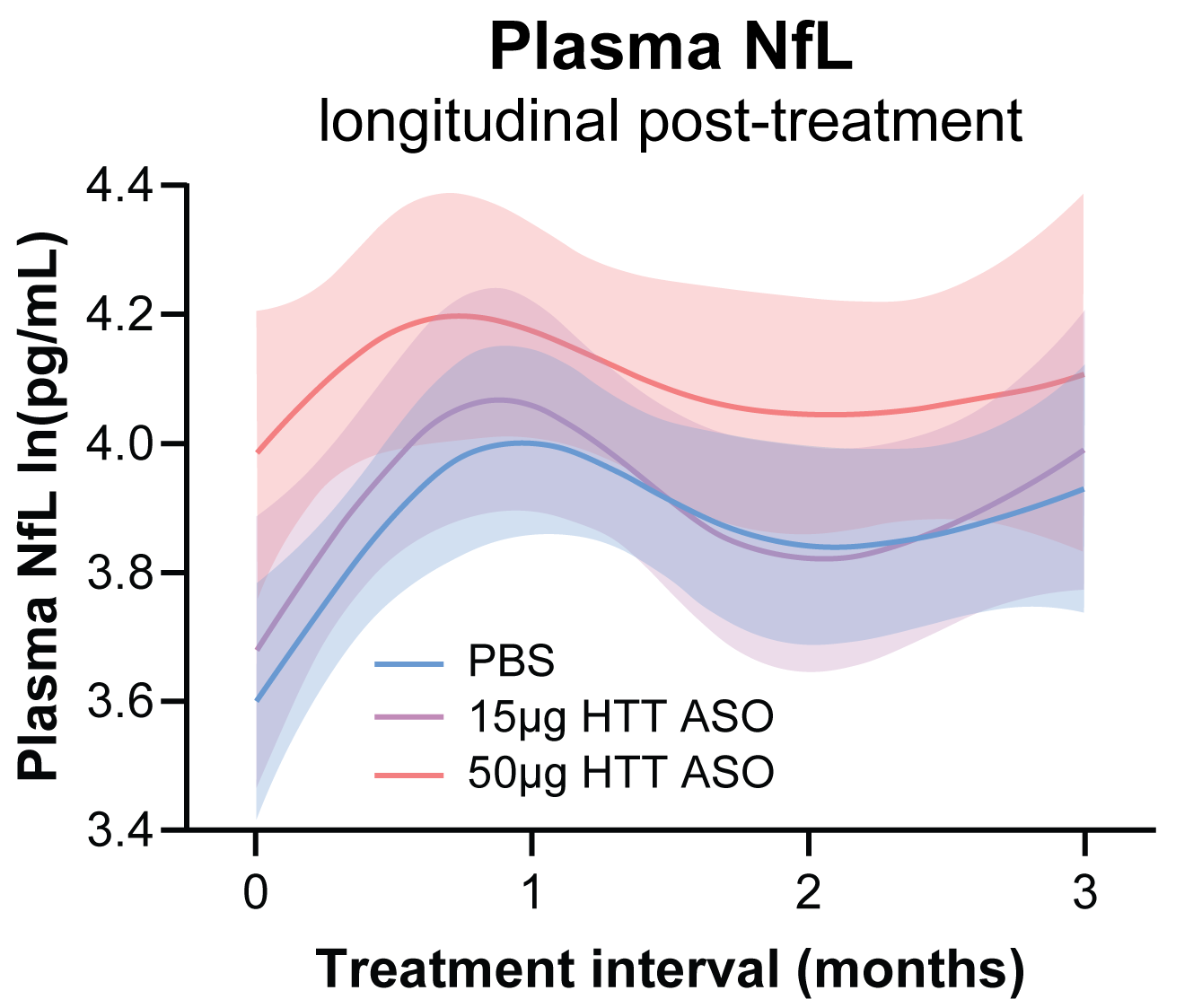
**

**Fig. S4.** **Plasma NfL concentrations following short duration mHTT lowering initiated at 2 months of age in the brains of YAC128 mice.** Longitudinal plasma NfL concentrations in YAC128 mice in response to treatment with either PBS, 15 µg HTT ASO or 50 µg HTT ASO (PBS: *n =* 29; 15 µg HTT ASO: *n =* 20; 50 µg HTT ASO: *n =* 13). Models show baseline values for all treatment conditions and were adjusted for time from treatment for each treatment condition and fit with a cubic spline. Solid lines denote the mean and shaded bands denote 95% CI for each treatment condition. Plasma and CSF NfL concentrations are natural log transformed.

ASO: antisense oligonucleotide, PBS: phosphate-buffered saline

**
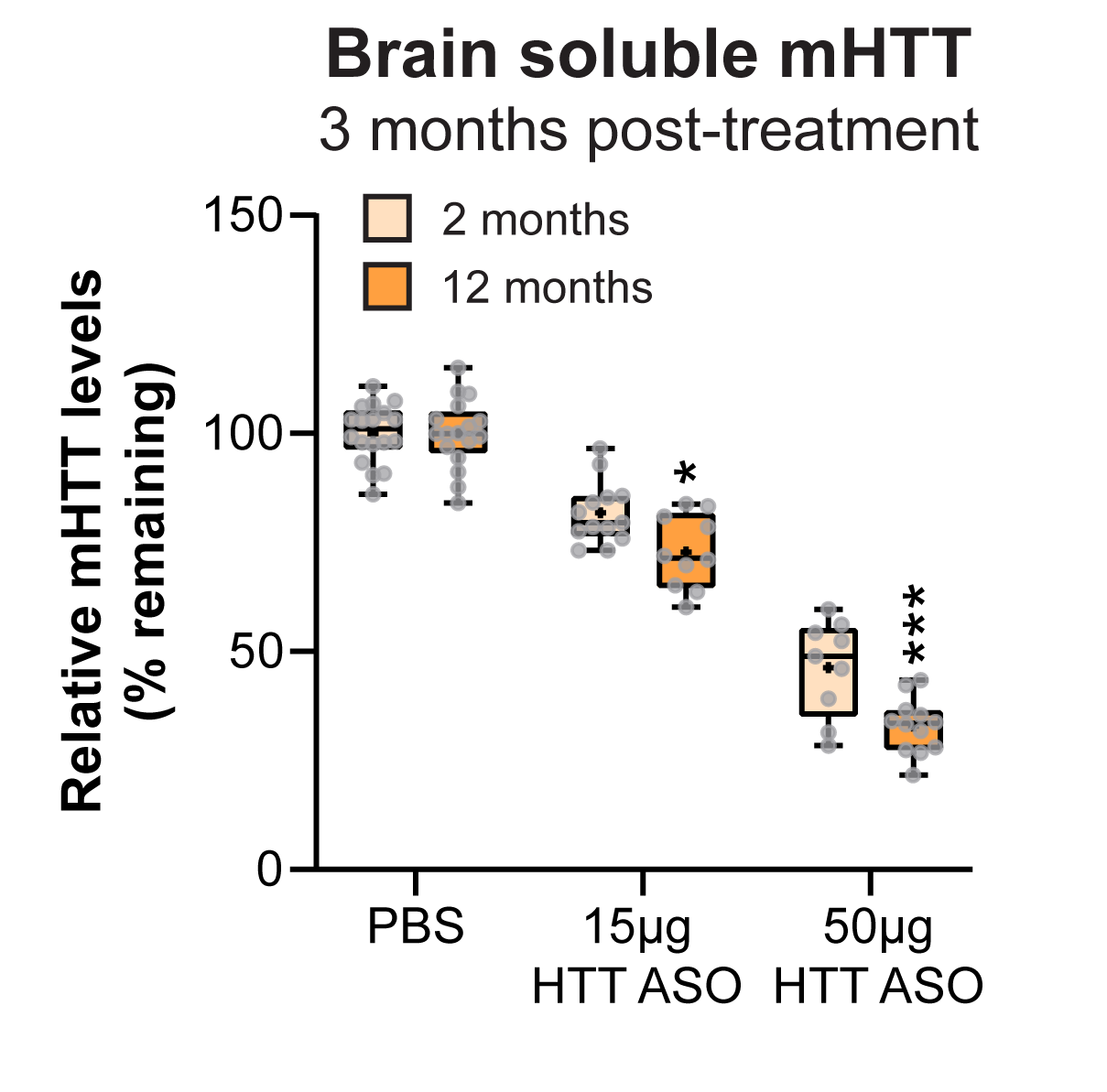
**

**Fig. S5.** **Short duration treatment with HTT ASO initiated at older ages leads to a higher magnitude of mHTT lowering in the brains of YAC128 mice.** Comparison of relative mHTT levels in the brain (mean mHTT from all brain regions for each animal) quantified by immunoblot following short duration treatment with PBS, 15 µg HTT ASO or 50 µg HTT ASO initiated at 2 (**Fig. 4c**) and 12 (**Fig. 5c**) months of age (2 months: *n =* 17 PBS, *n =* 10 15 µg HTT ASO, *n =* 10 50 µg HTT ASO; 12 months: *n =* 17 PBS, *n =* 10 15 µg HTT ASO, *n =* 12 50 µg HTT ASO. Two-way ANOVA: treatment *P* < 0.0001, age *P* < 0.0001, interaction *P* = 0.007. **P* = 0.023, ****P* = 0.0006). Levels of mHTT are shown relative to PBS values at each respective age. Boxes show the IQR, whiskers extend from minimum to maximum values, horizontal lines show the median, and crosses show the mean.

ASO: antisense oligonucleotide, IQR: interquartile range, PBS: phosphate-buffered saline

**
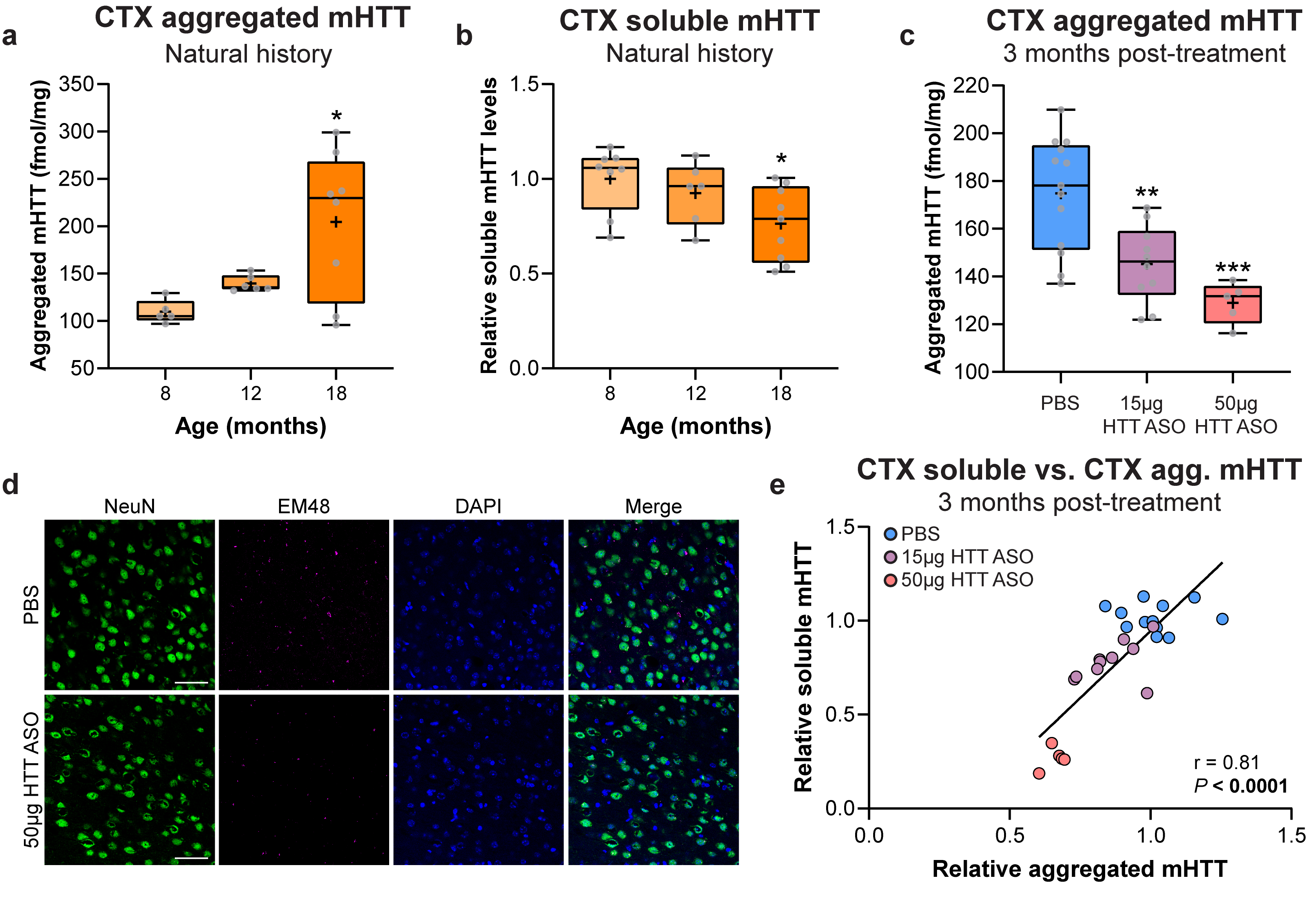
**

**Fig. S6.** **Short duration mHTT lowering initiated at 12 months of age reduces mHTT inclusion load in the brains of YAC128 mice. a, b** Concentrations of (**a**) aggregated mHTT (fmol/mg. 8 months: *n =* 5; 12 months: *n =* 6; 18 months: *n =* 8. One-way ANOVA: *P* = 0.011. 18 compared to 8 months: **P* = 0.013) and (**b**) relative soluble, full length mHTT in the cortex of YAC128 mice at 8, 12 and 18 months of age (8 months: *n =* 8; 12 months: *n =* 6; 18 months: *n =* 9. One-way ANOVA: *P* = 0.038. 18 compared to 8 months: **P* = 0.034). **c** Concentrations of aggregated mHTT in the CTX of YAC128 mice treated at 12 months with either PBS, 15 µg HTT ASO or 50µg HTT ASO and assessed at 3 months post-treatment (PBS: *n =* 13; 15 µg HTT ASO: *n =* 10; 50 µg HTT ASO: *n =* 5. One-way ANOVA: *P* = 0.0002. Compared to PBS: ***P* = 0.003, ****P* = 0.0004). Levels of mHTT at each time point are shown relative to 8-month values. Boxes show the IQR, whiskers extend from minimum to maximum values, horizontal lines show the median, and crosses show the mean. **d** IHC showing EM48 and NeuN labelling in the striatum of YAC128 mice treated with PBS or 50 µg HTT ASO at 12 months of age and imaged at 3 months post-treatment. Scale bar = 50 µm. **e** Correlation between relative aggregated and soluble mHTT (**Fig. 5c**) from paired CTX hemispheres from treated YAC128 mice (Pearson’s correlation: *r* = 0.81, *P* < 0.0001).

ASO: antisense oligonucleotide, CTX: cortex, IQR: interquartile range, PBS: phosphate-buffered saline

**
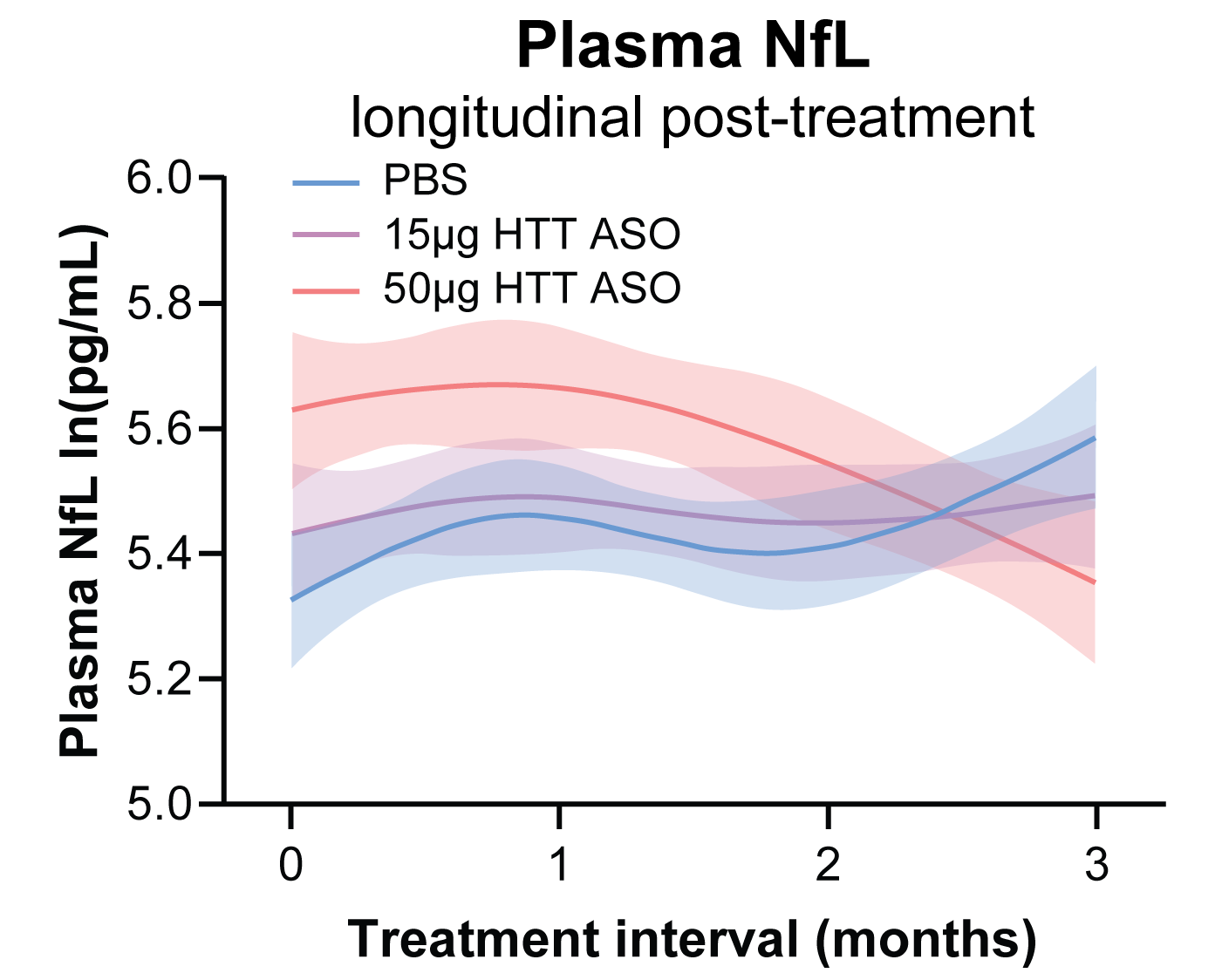
**

**Fig. S7.** **Plasma NfL concentrations following short duration mHTT lowering initiated at 12 months of age in the brains of YAC128 mice.** Longitudinal plasma NfL concentrations following treatment of YAC128 mice with either PBS, 15 µg HTT ASO or 50 µg HTT ASO (PBS: *n =* 20; 15 µg HTT ASO: *n =* 20; 50 µg HTT ASO: *n =* 12). Models show baseline values for all treatment conditions and were adjusted for time from treatment for each treatment condition and fit with a cubic spline. Solid lines denote the mean and shaded bands denote 95% CI for each treatment condition. Plasma and CSF NfL values are natural log transformed.

ASO: antisense oligonucleotide, PBS: phosphate-buffered saline

**
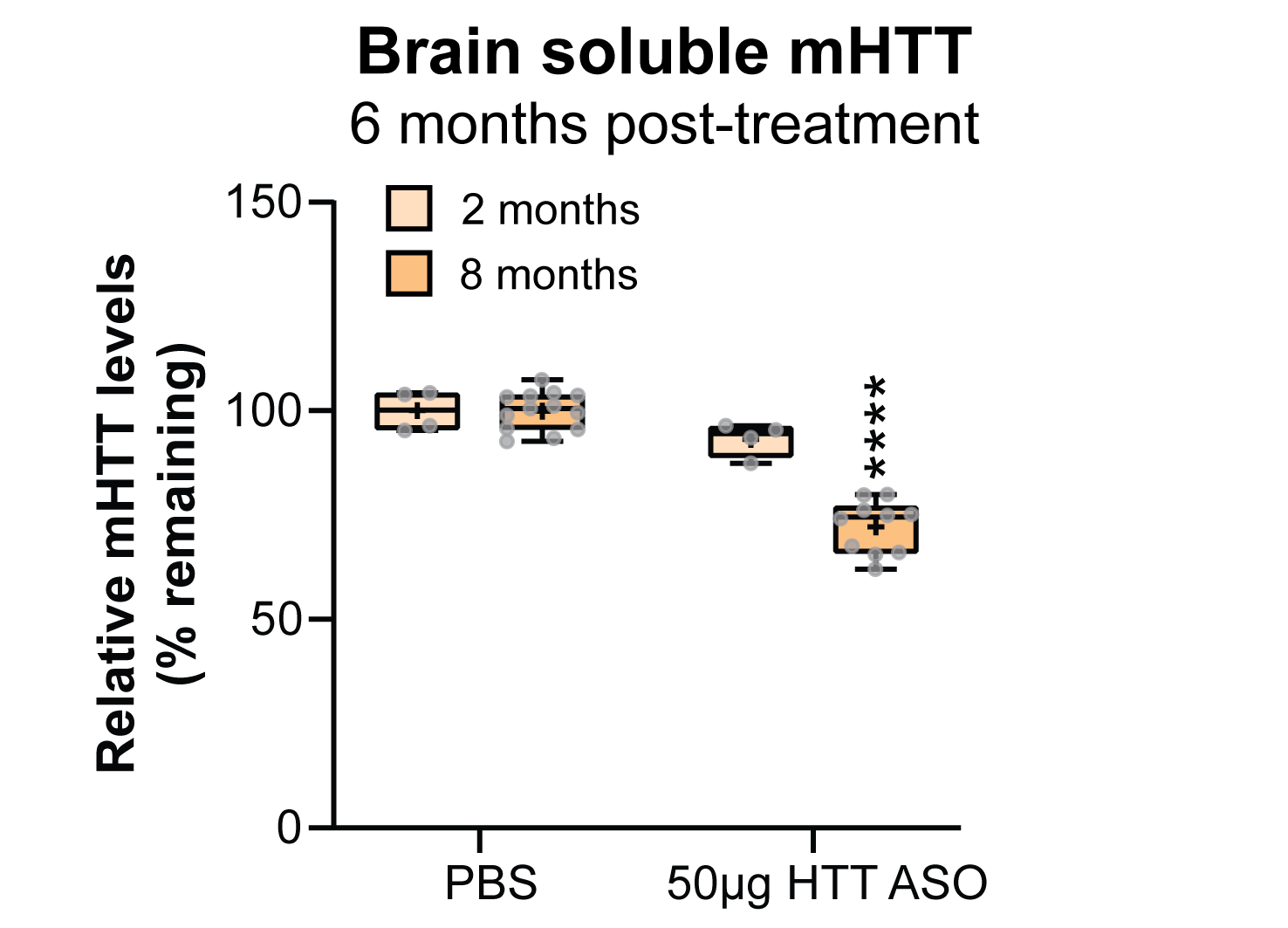
**

**Fig. S8.** **Long duration treatment with HTT ASO initiated at older ages leads to a higher magnitude of mHTT lowering in the brains of YAC128 mice.** Quantification of relative mHTT levels in the brain (mean mHTT from all brain regions for each animal) following long duration treatment with PBS or 50µg HTT ASO initiated at 2 (**Fig. S2)** and 8 (**Fig. 6c**) months of age (2 months: *n =* 4 PBS, *n =* 4 50 µg HTT ASO; 8 months: *n =* 13 PBS, *n =* 10 50 µg HTT ASO. Two-way ANOVA: treatment *P* < 0.0001, age *P* < 0.0001, interaction *P* < 0.0001. *****P* < 0.0001). Levels of mHTT are shown relative to PBS values at each respective time point. Boxes show the IQR, whiskers extend from minimum to maximum values, horizontal lines show the median, and crosses show the mean.

ASO: antisense oligonucleotide, IQR: interquartile range, PBS: phosphate-buffered saline


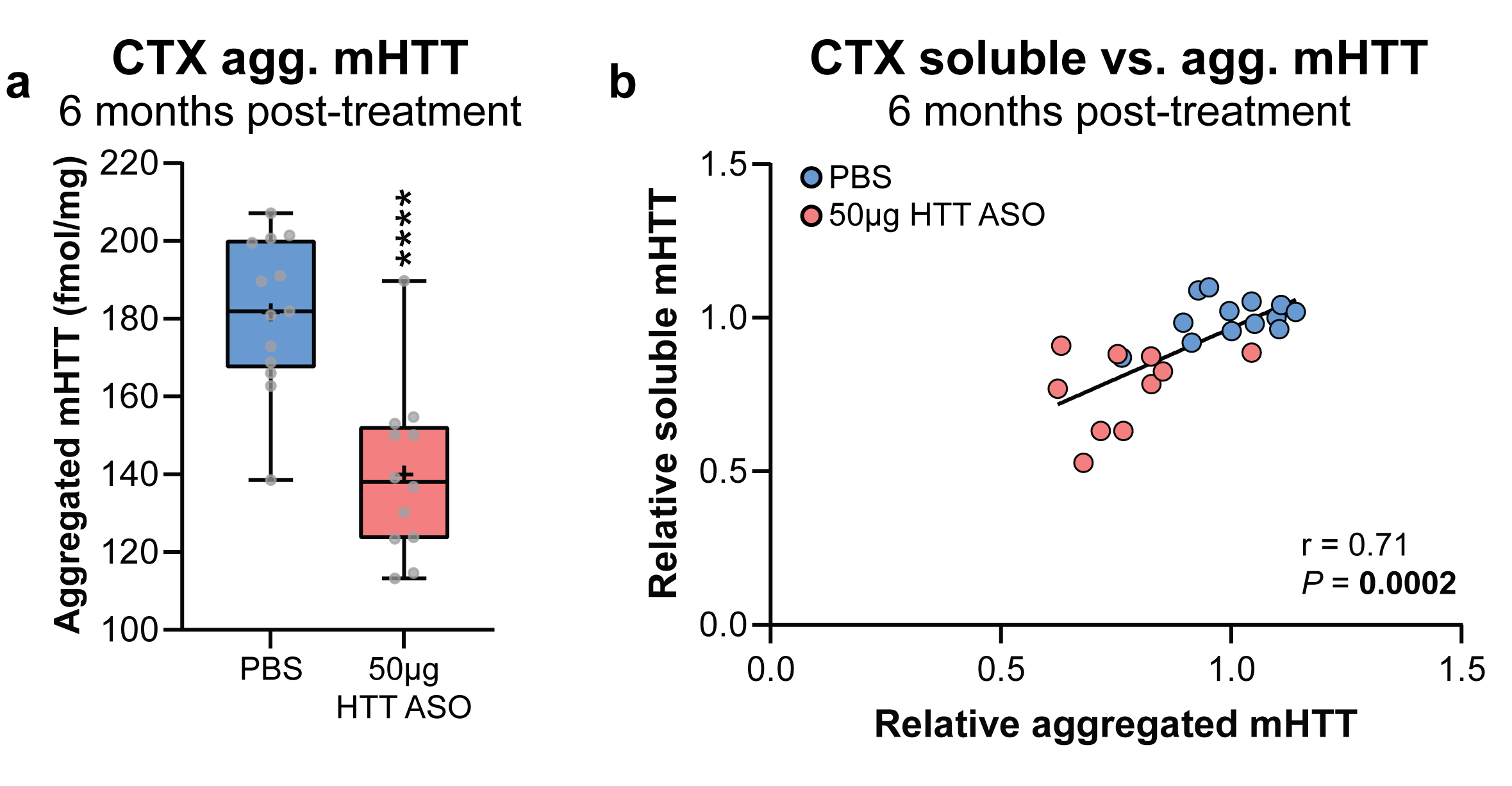


**Fig. S9.** **Long duration mHTT lowering initiated at 8 months of age reduces mHTT inclusion load in the brains of YAC128 mice. a** Concentrations of aggregated mHTT (fmol/mg) in the CTX of YAC128 mice treated at 8 months of age with either PBS or 50µg HTT ASO and assessed 6 months post-treatment (PBS: *n =* 13; 50µg HTT ASO: N= 12. Two-tailed t-test: *****P* < 0.0001). Boxes show the IQR, whiskers extend from minimum to maximum values, horizontal lines show the median, and crosses show the mean. **b** Correlation of relative aggregated and soluble mHTT (**Fig. 6c**) from paired CTX hemispheres from treated YAC128 mice (Pearson’s correlation: *r* = 0.71, *P* = 0.0002). ASO: antisense oligonucleotide, PBS: phosphate-buffered saline

**
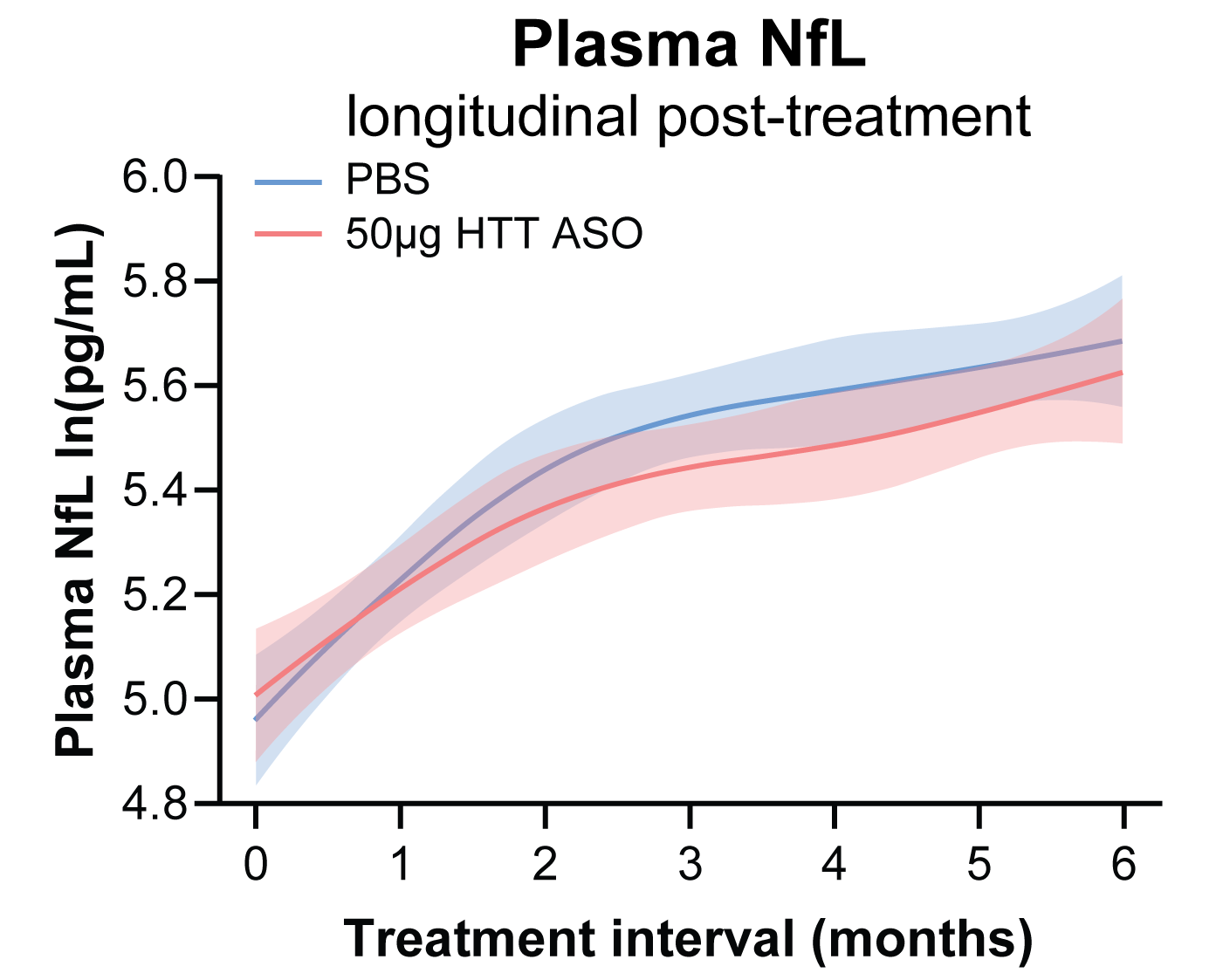
**

**Fig. S10. Plasma NfL concentrations following long duration mHTT lowering initiated at 8 months of age in the brains of YAC128 mice.** Longitudinal plasma NfL concentrations following treatment of YAC128 mice with either PBS or 50 µg HTT ASO (PBS: *n =* 13; 50 µg HTT ASO: *n =* 13). Models show baseline values for all treatment conditions and were adjusted for time from treatment for each treatment condition and fit with a cubic spline. Solid lines denote the mean and shaded bands denote 95% CI for each treatment condition. Plasma and CSF NfL values are natural log transformed. ASO: antisense oligonucleotide, PBS: phosphate-buffered saline

**
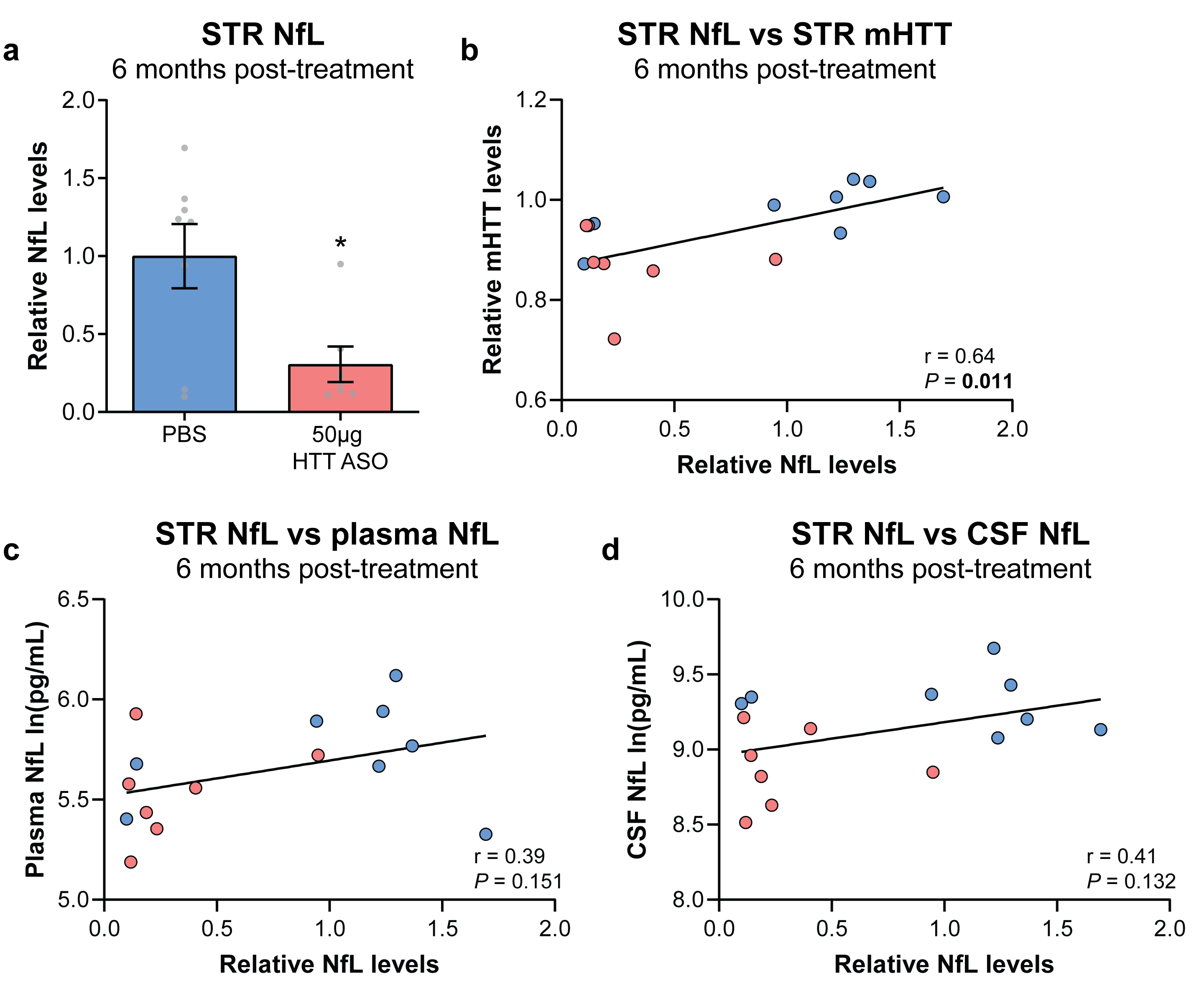
**

**Fig. S11. NfL protein levels are reduced in the striatum following long duration mHTT lowering in the brains of YAC128 mice. a** Quantification of relative NfL levels in the striatum following treatment with PBS or 50µg HTT ASO initiated at 8 months of age (PBS: *n =* 8, 50µg HTT ASO: *n =* 7. Unpaired t-test: P = 0.014). **c-e** Correlations between relative NfL levels in the striatum and (**b**) mHTT in the striatum (Pearson’s correlation: *r* = 0.64, *P* = 0.011), (**c**) plasma NfL (Pearson’s correlation: *r* = 0.39, *P* = 0.151), (**d**) CSF NfL (Pearson’s correlation: *r* = 0.41, *P* = 0.132).
