# Supplementary material for: Elevated plasma and CSF neurofilament light chain concentrations are stabilized in response to mutant huntingtin lowering in the brains of Huntington’s disease mice
Source: Transl Neurodegener. 2024 Oct 8;13:50. doi: 10.1186/s40035-024-00443-8 (PMC11460072; doi:10.1186/s40035-024-00443-8)
Supplement: Supplementary file 2 — Additional file 2: Immunoblot of NfL levels in the striatum of YAC128 mice at days 3 and 7 following intrastriatal injection with PBS or QA. [file 40035_2024_443_MOESM2_ESM.docx]

**Additional file 2**

**
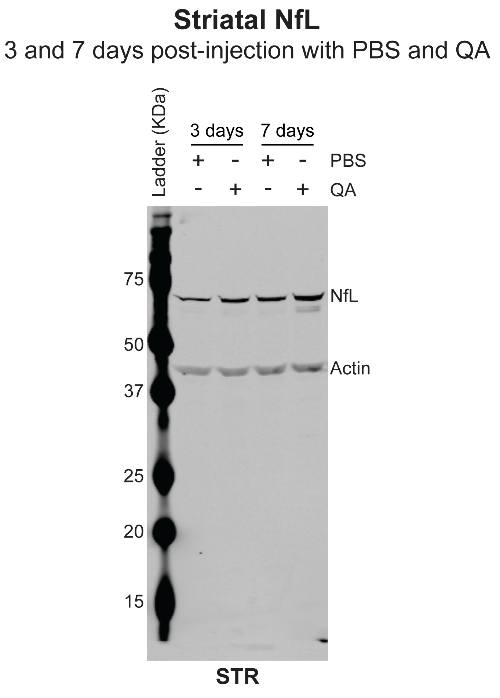
**

Immunoblot of NfL levels in the striatum of YAC128 mice at days 3 and 7 following intrastriatal injection with PBS or QA. Blot is representative of data presented in **Fig. 2h**.

STR: striatum

**
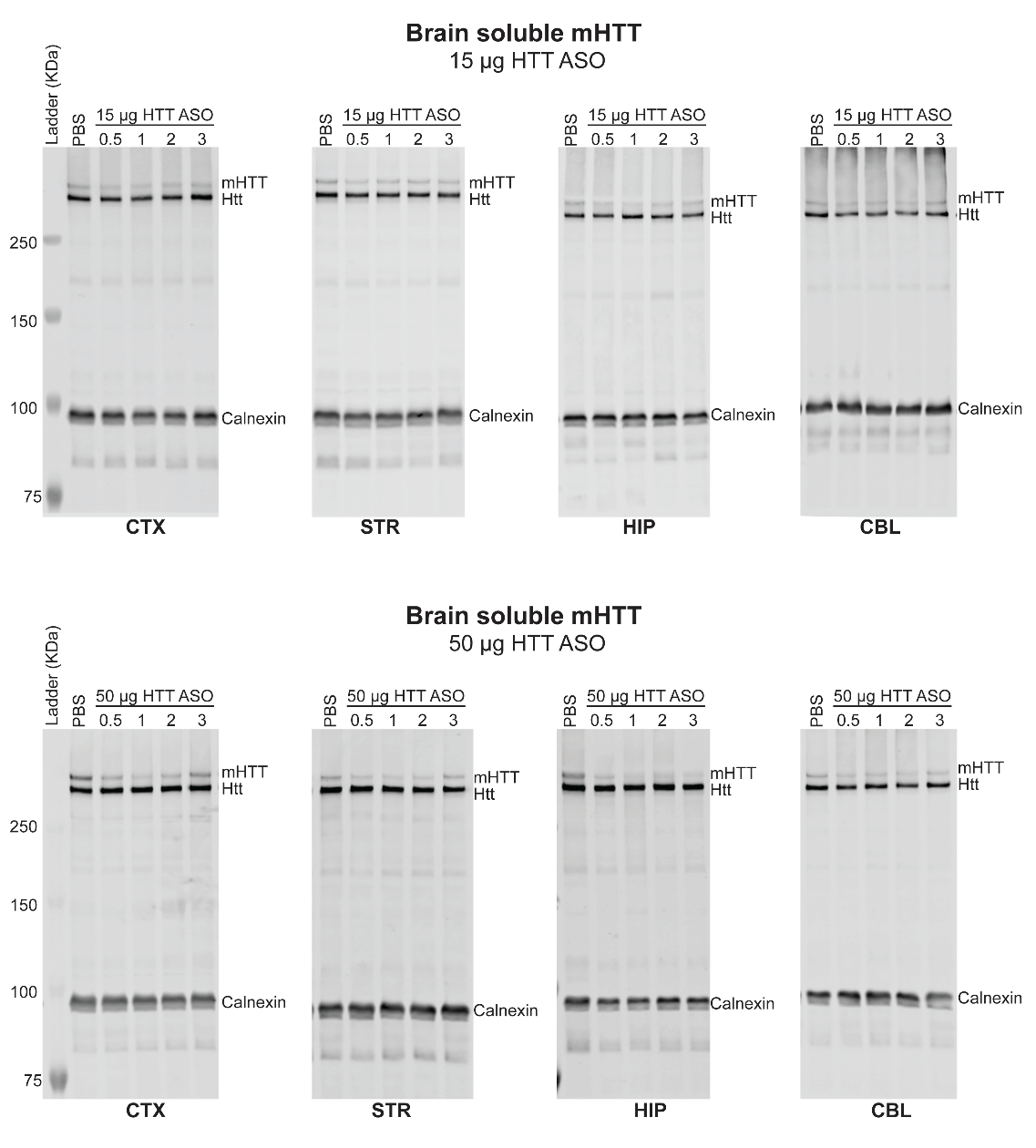
**

Immunoblots of mHTT levels in the brain of YAC128 mice at different time points up to 3 months following treatment with PBS, 15 µg HTT ASO or 50 µg HTT ASO. Blots are representative of data presented in **Fig. 3c-f**.

CTX: cortex, STR: striatum, HIP: hippocampus, CBL: cerebellum

**
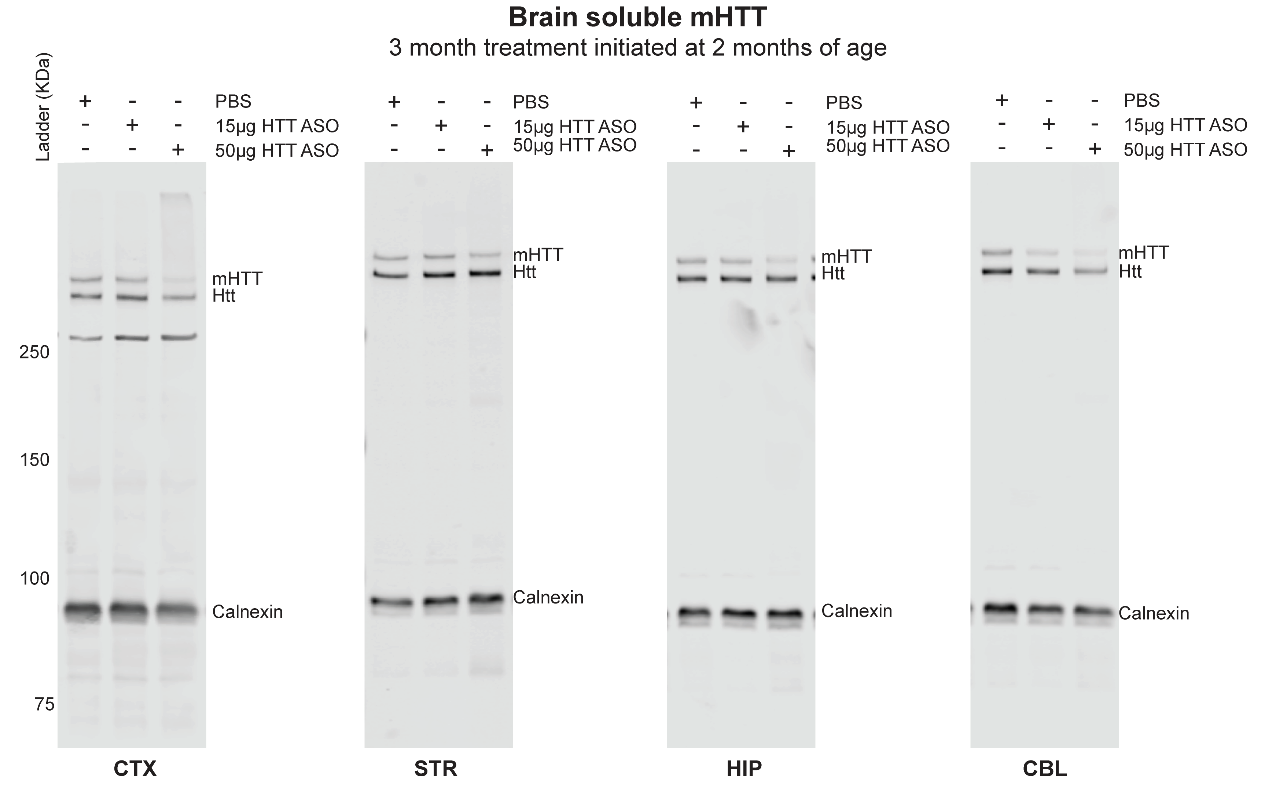
**

Immunoblots of mHTT levels in the brain of YAC128 mice following treatment with 15 µg and 50 µg HTT ASO initiated at 2 months of age and collected at 5 months of age (3 months post-treatment). Blots are representative of data presented in **Fig. 4c**.

CTX: cortex, STR: striatum, HIP: hippocampus, CBL: cerebellum

**
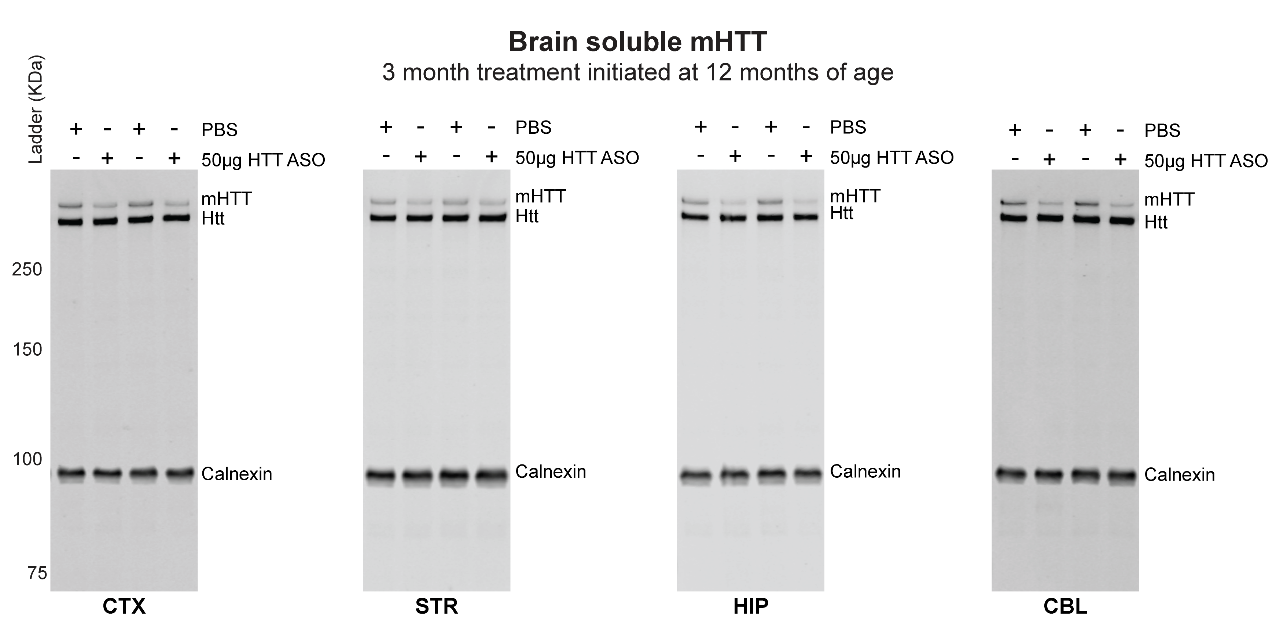
**

Immunoblots of mHTT levels in the brain of YAC128 mice following treatment with 50 µg HTT ASO initiated at 12 months of age and collected at 15 months of age (3 months post-treatment). Blots are representative of data presented in **Fig. 5c**.

CTX: cortex, STR: striatum, HIP: hippocampus, CBL: cerebellum

**
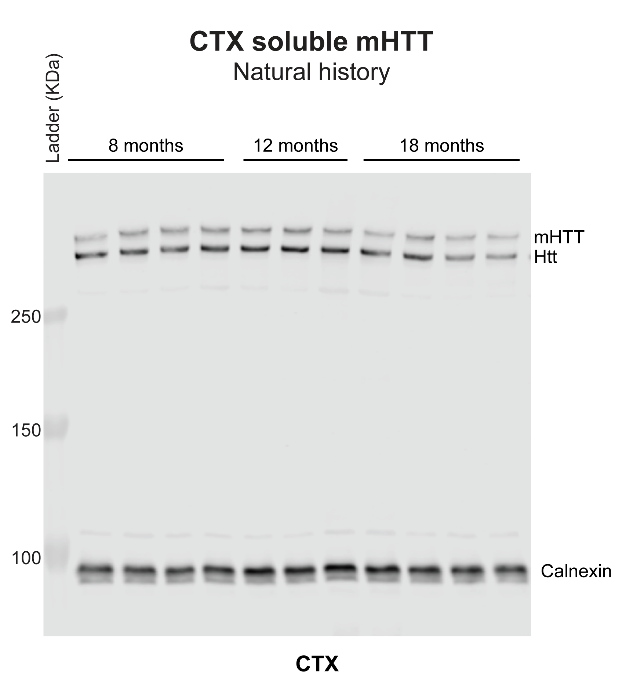
**

Immunoblot of mHTT levels in the cortex of YAC128 mice at 8, 12 and 18 months of age. Blot is representative of data presented in **Fig. S6b**.

CTX: cortex

**
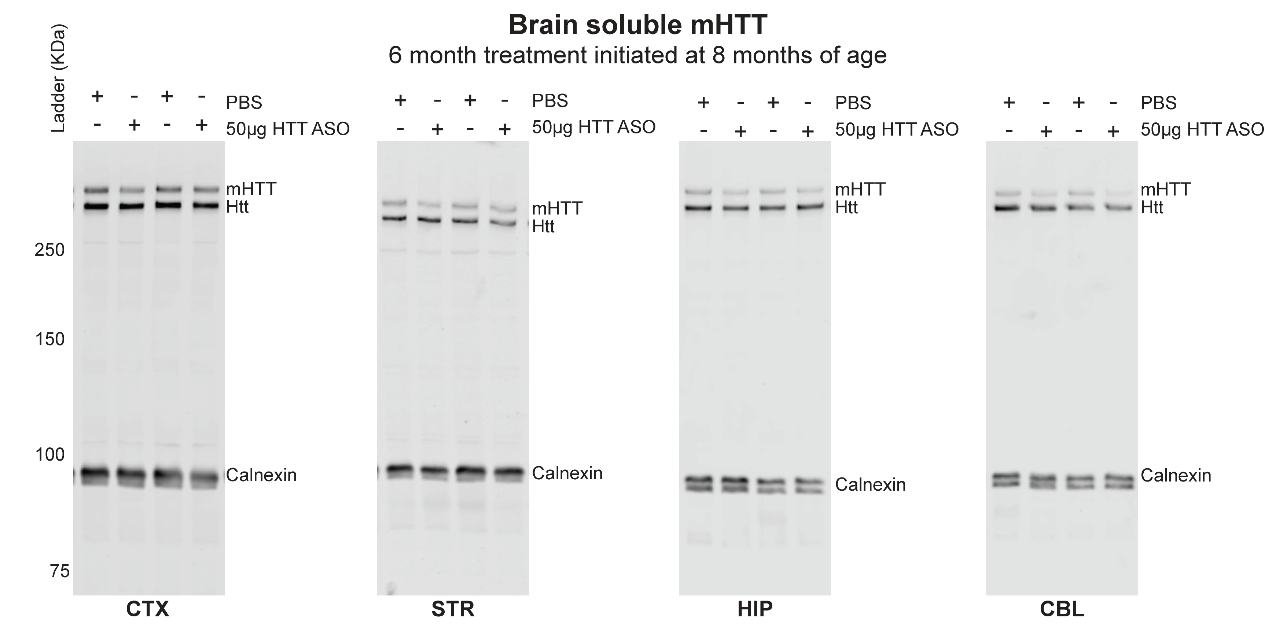
**

Immunoblots of mHTT levels in the brain of YAC128 mice following treatment with PBS or 50 µg HTT ASO initiated at 8 months of age and collected at 14 months of age (6 months post-treatment). Blots are representative of data presented in **Fig. 6c**.

CTX: cortex, STR: striatum, HIP: hippocampus, CBL: cerebellum


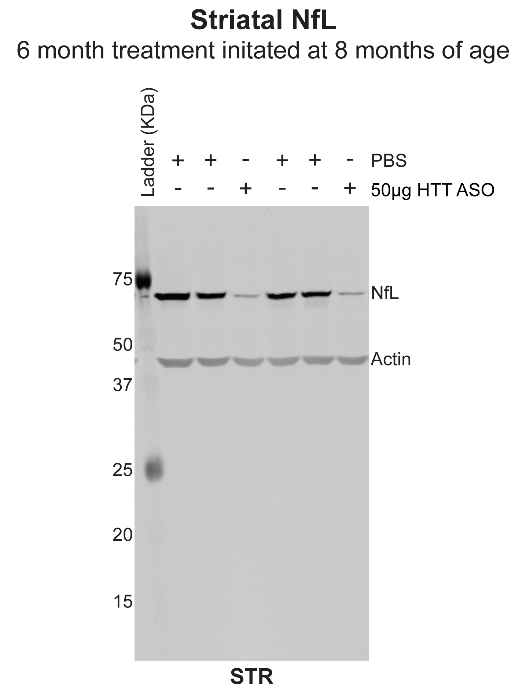


Immunoblot of NfL levels in the striatum of YAC128 mice following treatment with PBS or 50 µg HTT ASO initiated at 8 months of age and collected at 14 months of age (6 months post-treatment). Blot is representative of data presented in **Fig. S11a**.

STR: striatum
